# Autophagy initiation by ULK complex assembly on ER tubulovesicular regions marked by ATG9 vesicles

**DOI:** 10.1038/ncomms12420

**Published:** 2016-08-11

**Authors:** Eleftherios Karanasios, Simon A. Walker, Hanneke Okkenhaug, Maria Manifava, Eric Hummel, Hans Zimmermann, Qashif Ahmed, Marie-Charlotte Domart, Lucy Collinson, Nicholas T. Ktistakis

**Affiliations:** 1 Signalling Programme, The Babraham Institute, Cambridge CB22 3AT, UK; 2 Carl Zeiss Microscopy GmbH, Munich 81379, Germany; 3 The Francis Crick Institute, London NW1 1AT, UK

## Abstract

Autophagosome formation requires sequential translocation of autophagy-specific proteins to membranes enriched in PI3P and connected to the ER. Preceding this, the earliest autophagy-specific structure forming *de novo* is a small punctum of the ULK1 complex. The provenance of this structure and its mode of formation are unknown. We show that the ULK1 structure emerges from regions, where ATG9 vesicles align with the ER and its formation requires ER exit and coatomer function. Super-resolution microscopy reveals that the ULK1 compartment consists of regularly assembled punctate elements that cluster in progressively larger spherical structures and associates uniquely with the early autophagy machinery. Correlative electron microscopy after live imaging shows tubulovesicular membranes present at the locus of this structure. We propose that the nucleation of autophagosomes occurs in regions, where the ULK1 complex coalesces with ER and the ATG9 compartment.

## Abstract (web summary)

The ULK1 complex is required during autophagosome nucleation, but where autophagic membranes initiate is unknown. Here the authors use super-resolution microscopy to propose that autophagosomes originate from tubulovesicular structures in the ER that align with ATG9 vesicles and recruit ULK1.

## Graphical abstract

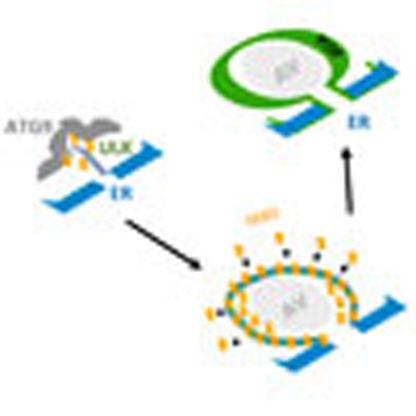

Autophagy is the membrane trafficking pathway that delivers intracellular material for degradation to lysosomes via *de novo* formation of double-membrane vesicles, the autophagosomes. Cells activate autophagy in response to nutrient limitation or accumulation of damaged proteins and organelles, and as a result they recycle building blocks generated in lysosomes into new macromolecules and sub-cellular structures^1^. Unsurprisingly, autophagy has implications for ageing and associated diseases, such as neurodegeneration, inflammation and cancer^2^,^3^,^4^,^5^. Autophagy also underpins physiological functions, such as development, cell differentiation and immunity^6^.

Autophagosome formation requires a protein machinery to act upon a membrane source, organize it into a flat sheet, expand it and finally fuse its extremities to enclose the cytosolic cargo. Yeast genetics have identified >30 autophagy-related (Atg) genes encoding the core autophagy machinery, most of which are conserved in mammals^3^,^7^. This protein machinery is organized into functional complexes that carry out the steps of autophagosome formation^3^. In brief, scarcity of amino acids inactivates mechanistic target of rapamycin (mTORC1) and releases the repression of a functional complex, including the protein kinase ULK1, which then translocates to membranes (initiation step). ULK1 activates the functional complex, including the lipid kinase VPS34, stimulating the synthesis of phosphatidylinositol 3-phosphate (PI3P) and the formation of an omegasome (nucleation step). The omegasomes are membrane platforms in contact with endoplasmic reticulum (ER), where the remaining core machinery is recruited. This includes vesicles of ATG9, the only transmembrane autophagy protein, and two conjugation systems, ultimately leading to the covalent attachment of the small ubiquitin-like protein LC3 to phosphatidylethanolamine (lipidation). LC3, the signature protein of autophagosomes, promotes the expansion of the autophagosomal membrane (also known as isolation membrane, elongation step), and its closure and fusion with the lysosome (maturation step).

The characterization of the membrane source that drives the nucleation and elongation of autophagosomes has proven so far to be more elusive. It is generally accepted that more than one membrane sources are likely to be involved in the different steps of autophagosome formation, including the ER, mitochondria, mitochondria-associated membranes, the Golgi, the plasma membrane and recycling endosomes^8^,^9^,^10^,^11^,^12^,^13^,^14^. Efforts to identify this membrane source have focused on two fronts: the co-localization of the autophagic machinery with pre-existing organelles and the characterization of the membrane compartment that hosts ATG9, the only transmembrane autophagy protein. Among the pre-existing organelles, autophagosomes induced by amino-acid starvation emerge adjacent to ER; however, the mechanistic contribution of this arrangement has remained unknown^8^,^12^,^15^,^16^,^17^,^18^. One possibility is that the autophagic machinery associates with two ER-associated membrane compartments: the ER exit sites (ERES) and the ER–Golgi intermediate compartment (ERGIC)^19^,^20^,^21^,^22^,^23^,^24^. The ERES are sites where proteins trafficked to Golgi are packaged into coat protein complex I (COPII)-coated carriers, creating an adjacent collection of vesicular–tubular structures that constitutes the ERGIC^25^. Of note, the Rab GTPase Rab1/Ypt1, which is required for the ER-to-Golgi trafficking, also promotes autophagy^26^,^27^,^28^,^29^,^30^. A second possibility is that the ER coordinates the redistribution of the ATG9 compartment during autophagy^14^,^31^,^32^. Interestingly, Ypt1 binds to Atg9 (refs 29, 33), one of the first proteins recruited at the pre-autophagosomal structure (PAS), promoting the recruitment of downstream proteins^14^,^34^. Moreover, the mammalian ATG9 colocalizes at the some point of its life cycle with the ULK1 complex^13^,^32^.

The recruitment of the ULK1 complex confers to the earliest autophagy-specific structure forming *de novo* its autophagic character. Characterizing the provenance and mode of formation of this structure though has proven to be challenging: it is a short-lived intermediate that has not yet acquired the characteristic double-membrane crescent shape identified by electron microscopy. Herein, we take a comprehensive imaging-based approach to address where the ULK1 complex nucleates autophagosome formation. We find that both ERES/ERGIC and ATG9 are functionally important for the nucleation of autophagosomes, though ATG9 vesicles are more frequently observed in association with the earliest detectable autophagy structures. We develop for the first time a protocol for direct Stochastic Optical Reconstruction Microscopy (dSTORM) of autophagy-related structures and we observe the autophagic machinery in combination with the ERES/ERGIC and ATG9 compartments at sub-diffraction resolution. We find that ATG13 decorates a unique compartment that associates with small vesicles of ATG9 or ERGIC, but not with the conventional ERES. Using correlative light and electron microscopy (CLEM), we find that the ATG13 structures correspond to a tubulovesicular compartment surrounded by ER and mitochondria. We propose that new autophagosomes nucleate in regions, where the ULK1 complex coalesces with the ER and vesicles of ATG9.

## Results

### ERES/ERGIC contributes to autophagosome nucleation

**Figure 1:**
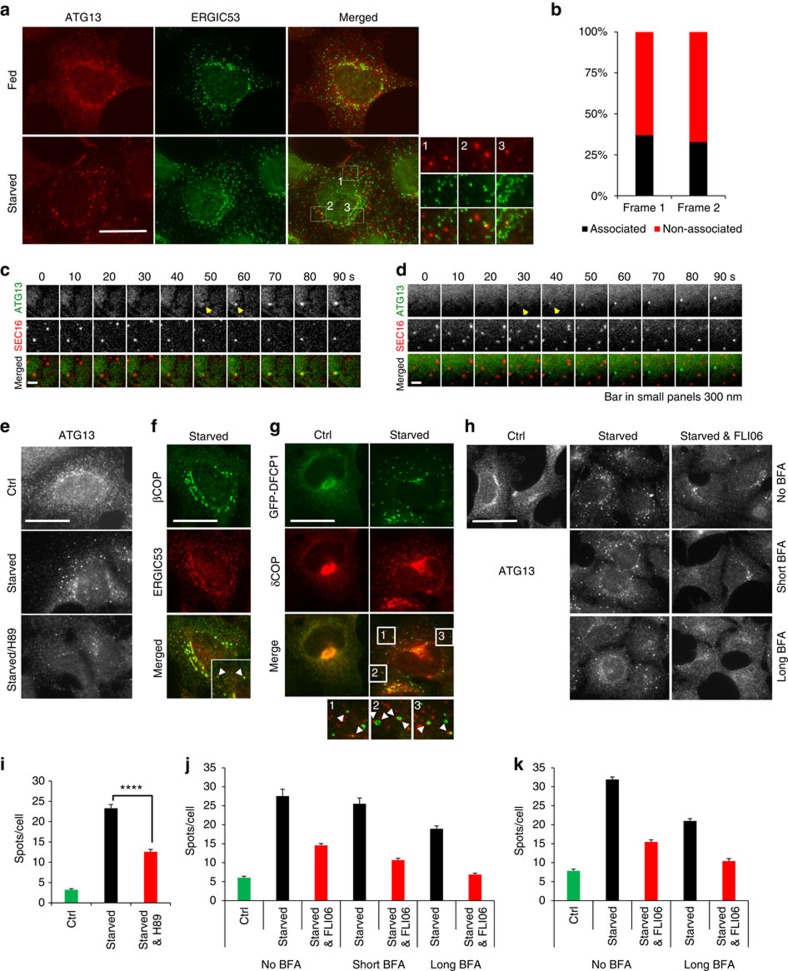
ER exit promotes the formation of ATG13 puncta. (**a**) HEK293 cells were fed or starved for 1 h, immunolabelled for ATG13 and ERGIC53, and imaged by wide-field microscopy. (**b**) Values are ATG13 particles in **c**,**d** associating with SEC16 particles in the first two frames from their emergence. From analysis of 73 montages. (**c**,**d**) Wide-field live-cell imaging of starved HEK293 cells expressing stably GFP-ATG13 and transiently mCherry-SEC16. Representative montages of ATG13 particles forming in association with SEC16 (**c**) or not (**d**) are shown. Arrowheads point at the ATG13 particles in the first two frames from their emergence, the same that were used for the analysis in **b**. (**e**) HEK293 cells were fed or starved for 1 h, treated with 50 μM H89 in the last 30 min, immunolabelled for ATG13 and imaged by wide-field microscopy. (**f**) HEK293 cells were starved for 1 h, immunolabelled for βCOP and ERGIC53 and imaged by wide-field microscopy. (**g**) HEK293 cells stably expressing GFP-DFCP1 were fed or starved, immunolabelled for δCOP and imaged by wide-field microscopy. Arrowheads in insets point at COPI particles adjacent to DFCP1 puncta or rings. (**h**) HEK293 cells were pre-treated with 3 μg ml^−1^ BFA for 3 h (long BFA) or for 30 min (short BFA) and for 30 min with 100 μM FLI06, then starved for 1 h in the presence or absence of BFA and FLI06, immunolabelled for ATG13 and imaged by confocal laser scanning microscopy. (**i**) Values are means±s.e.m. puncta of ATG13 per cell in **e**, for at least five different fields with 15–30 cells each. (**j**) Values are means±s.e.m. puncta of ATG13 per cell in **h**, for at least five different fields with 15–30 cells each. (**k**) Values are means±s.e.m. puncta of ATG13 per cell in an independent experiment, for at least five different fields with 15–30 cells each. Significance levels were determined with unpaired *t*-tests. ****P=0.0001%. Bar in **a**,**e**–**h** corresponds to 10 μm. Bar in **c**–**e** corresponds to 300 nm.

Autophagosomes induced by amino-acid starvation in HEK293 cells form on ER-associated PI3P-rich structures called omegasomes^8^. It was recently proposed that the site of autophagosome nucleation on the ER is ERES^22^,^35^. We hypothesized that if ERES/ERGIC are the platform of autophagosome nucleation they should colocalize with puncta of the ULK1 complex, the earliest autophagy-specific structure. We used as a surrogate for the ULK1 complex ATG13, which is an important stable component of the complex involved exclusively in autophagy that has been extensively characterized (refs 16, 36, 37). Under autophagy-inducing conditions only a fraction of ATG13 puncta associated clearly with ERGIC53 (ERGIC marker; Fig. 1a) or SEC23 and SEC23IP (ERES markers; Supplementary Fig. 1). We reasoned that ATG13 might associate with ERES/ERGIC only during the nucleation step. However, in live cells only one third of the GFP-ATG13 particles emerged in association with mCherry-SEC16 (ERES marker^38^; Fig. 1b–d), suggesting that ERES/ERGIC can only partially account as platform for autophagosome nucleation.

The canonical function of ERES is the COPII-dependent packaging of proteins into vesicles and their trafficking to ERGIC on their way to Golgi^39^. Trafficking from ERES is also required for the elongation of autophagosomes^22^,^23^. However, these studies did not address if ER exit contributes to the upstream nucleation step of autophagosome formation. Blocking ER exit with the drug H89^23^, we found that under amino-acid starvation the ATG13 puncta decreased by ∼50% (Fig. 1e,i). However, H89 markedly altered cellular morphology and reduced phosphorylation of cellular proteins (Supplementary Fig. 2), suggesting that it may cause toxicity hence compromising autophagosome formation indirectly. To preclude this possibility, we also blocked ER exit with FLI06, which prevents the loading of cargo to the COPII complex thus inhibiting general secretion just before the ER exit^40^. FLI06 decreased ATG13 puncta to a similar extent with H89 (∼50%; Fig. 1h,j,k), corroborating that ER exit contributes to autophagosome nucleation.

Blocking ER exit attenuated, but did not abolish the formation of ATG13 puncta, suggesting that additional machineries may contribute to the process. One such machinery is the coat protein complex I (COPI) that mediates retrograde trafficking from Golgi to ERGIC^41^. COPI was recently linked with autophagosome maturation^42^. Under autophagy-inducing conditions a pool of COPI relocated to ERGIC (Fig. 1f), and often localized in close proximity to omegasomes (Fig. 1g), suggesting that COPI may also contribute to autophagosome nucleation. Indeed, brefeldin A (BFA), which blocks COPI-mediated trafficking to ERGIC^43^, decreased ATG13 puncta during amino-acid starvation in a time-dependent manner (Fig. 1h,j,k). Importantly, blocking bidirectional trafficking to ERGIC with combined BFA and FLI06 treatment further decreased ATG13 puncta to levels similar with non-starved cells (Fig. 1h,j,k), suggesting that both trafficking pathways contribute to autophagosome nucleation.

### COPI contributes to autophagosome elongation and maturation

**Figure 2:**
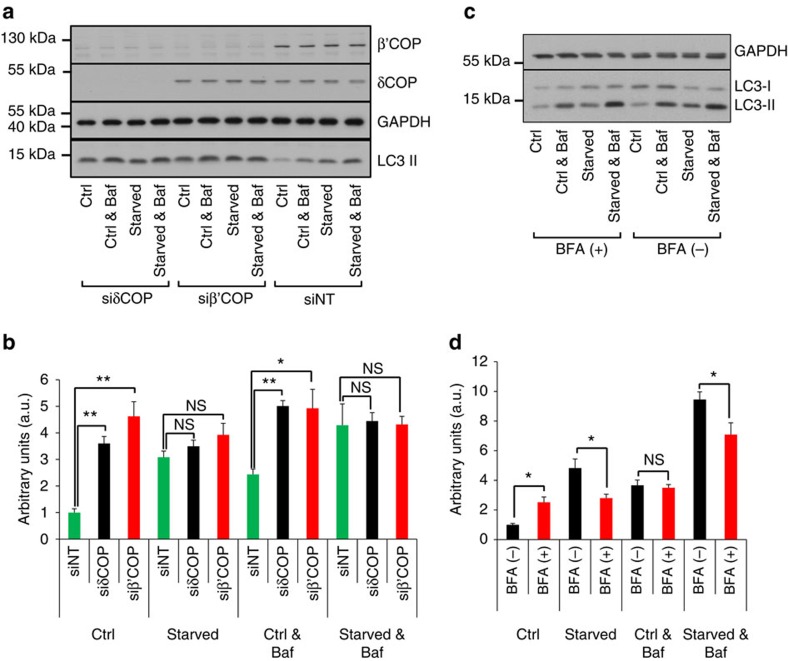
COPI complex promotes lipidation of LC3. (**a**) HEK293 cells were transfected with siRNA targeting δCOP (siδCOP), β'COP (siβ'COP) or non-targeted (siNT), starved in the presence or absence of 0.1 μM bafilomycin and lysed. Lysates were assessed by western blotting using antibodies against LC3, β'COP, δCOP and GAPDH. Note that siRNA depletion of δCOP decreases the stability of β'COP. (**b**) Western blots from **a** were quantitated and values of LC3 II normalized to GAPDH and then to the siNT at control conditions are shown as means±s.e.m. From five independent experiments. One arbitrary unit (a.u.) equals the average of LC3 II in fed cells. (**c**) HEK293 cells were pre-treated for 3 h with 3 μg ml^−1^ BFA, starved for 1 h in the presence or absence of BFA and bafilomycin and lysed. Lysates were assessed by western blotting using antibodies against LC3 and GAPDH. (**d**) Western blots from **c** were quantitated and values of LC3 II normalized to GAPDH and then to the control conditions in the absence of BFA are shown as means±s.e.m. From six independent experiments. One arbitrary unit (a.u.) equals the average of LC3 II in fed cells. Significance levels were determined with repeated-measures analysis of variance followed by Holm–Sidak's multiple comparison tests. NS non significant; *P=0.05%; **P=0.01%.

Our results suggesting that COPI contributes to the early steps of autophagosome formation (Fig. 1) expand on recent findings that COPI contributes to autophagosome maturation^42^, but is not required for the steps upstream of LC3 lipidation (nucleation and elongation)^23^. To examine this further, we assessed the contribution of COPI in LC3 lipidation (formation of LC3-II), which is an obligatory step for the expansion of the autophagosomal membrane. In fed cells, when we knocked down different subunits of COPI (either δCOP or β'COP), we found that LC3-II levels increased (compare ctrl\siδCOPl and ctrl\siβ'COPl with ctrl\siNT in Fig. 2a,b). Bafilomycin A, which prevents LC3-II degradation in lysosomes, did not further increase LC3-II significantly (compare ctrl\siδCOP with ctrl&baf\siδCOP and ctrl\siβ'COP with ctrl&baf\siβ'COP in Fig. 2a,b), confirming that COPI contributes to autophagosome maturation. Importantly, combination of COPI knock down (which blocks LC3 II degradation) with amino-acid starvation for 1 h (that creates a pulse of increased LC3 lipidation), did not further increase LC3-II significantly (compare ctrl\siδCOP with starved\siδCOP and ctrl\siβ'COP with starved\siβ'COP in Fig. 2a,b), suggesting that COPI also contributes to LC3 lipidation.

We also examined if inhibition of COPI by BFA treatment has the same effect on LC3 lipidation. In fed cells, similarly with the knock down of COPI, BFA increased LC3-II (compare ctrl\BFA(−) with ctrl\BFA(+) in Fig. 2c,d), suggesting either increased LC3 lipidation or impaired LC3-II degradation. Combination of BFA with bafilomycin A in fed cells did not further increase LC3-II significantly (compare ctrl\BFA(+) with ctrl&baf\BFA(−) and ctrl&baf\BFA(+) in Fig. 2c,d), suggesting that only LC3 degradation may be compromised. However, combination of BFA treatment with amino-acid starvation did not further increase LC3-II (compare ctrl\BFA(+) with starved\BFA(+) in Fig. 2c,d), suggesting that in starved cells COPI may also contribute to LC3 lipidation. Moreover, combination of BFA treatment and amino-acid starvation with bafilomycin A attenuated the increase of LC3-II (compare starved&baf\BFA(−) with starved&baf\BFA(+) in Fig. 2c,d), further supporting that under amino-acid starvation COPI contributes to LC3 lipidation.

### COPI facilitates the assembly of the lipidation machinery

**Figure 3:**
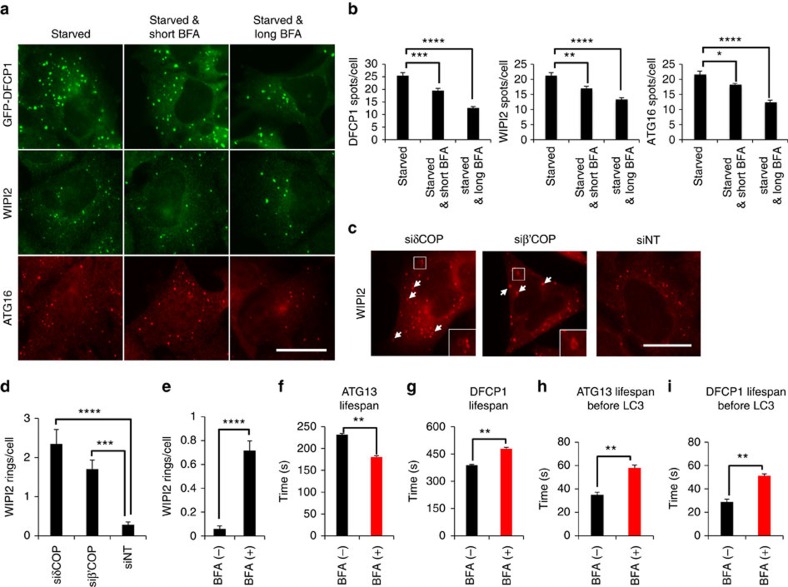
COPI complex promotes elongation of autophagosomes. (**a**) HEK293 cells stably expressing GFP-DFCP1 or the parental cell line were pre-treated for 3 h with 3 μg ml^−1^ BFA (long BFA), starved for 1 h in the presence of BFA and the parental cell line was immunolabelled for WIPI2 or ATG16. Cells were imaged by wide-field microscopy. Short BFA corresponds to non-pre-treated cells. Bar corresponds to 10 μm. (**b**) Values are means±s.e.m. of DFCP1, WIPI2 and ATG16 spots per cell in **a**, from at least six fields with 5–10 cells each. (**c**) HEK293 cells were transfected with δCOP (siδCOP), β'COP (siβ'COP) or non-targeted (siNT) siRNA, starved, immunolabelled for WIPI2 and imaged by wide-field microscopy. Arrows point at WIPI2 ring-like structures. Bar corresponds to 10 μm. (**d**) Values are means±s.e.m. of WIPI2 rings per cell in **c**, for 10 different fields with 5–15 cells each. (**e**) HEK293 cells were pre-treated for 3 h with 3 μg ml^−1^ BFA, starved, immunolabelled for WIPI2 and imaged by wide-field microscopy. Values are means±s.e.m. of WIPI2 rings per cell, for 10 different fields with 5–15 cells each. (**f**,**g**) HEK293 cells stably expressing GFP-ATG13 (**f**) or GFP-DFCP1 (**g**) were pre-treated for 3 h with 3 μg ml^−1^ BFA, starved and live-imaged in the presence of BFA by wide-field microscopy. The lifespan of ATG13 and DFCP1 particles was quantitated. Values are means±s.e.m. of ATG13 or DFCP1 particle lifespan, from 60 and 30 montages, respectively. (**h**,**i**) HEK293 cells expressing stably GFP-ATG13 (**h**) or GFP-DFCP1 (**i**) and transiently CFP-LC3 were pre-treated for 3 h with 3 μg ml^−1^ BFA, starved and live-imaged in the presence of BFA by wide-field microscopy. The lifespan of ATG13 and DFCP1 particles before the appearance of LC3 was quantitated. Values are means±s.e.m. of ATG13 or DFCP1 particle lifespan before the appearance of LC3, from 20 and 26 montages, respectively. Significance levels were determined with unpaired *t*-tests. *P=0.05%; **P=0.01%; ***P=0.001%; ****P=0.0001%.

We further explored how COPI may promote LC3 lipidation by examining the localization of several autophagic markers that function upstream of the lipidation of LC3. We found that BFA decreased the number of puncta formed by DFCP1, WIPI2 and ATG16 (Fig. 3a,b), suggesting that COPI inhibition may compromise the recruitment of the autophagic machinery. Indeed, depletion of COPI in starved cells misdirected WIPI2 to ring-like structures that did not associate with autophagosomes (Fig. 3c; Supplementary Fig. 3). When compared with control cells, these structures were observed up to 8.5 times more frequently in COPI-depleted cells (compare siδCOP with siNT in Fig. 3d) and 12 times more frequently in BFA-treated cells (Fig. 3e). We also quantitated the dynamics of LC3 accumulation to puncta hosting the early autophagy proteins ATG13 or DFCP1. BFA treatment shortened the lifespan of ATG13 particles (20%; Fig. 3f), but prolonged the lifespan of DFCP1 particles (over 20%; Fig. 3g). Importantly, COPI inhibition delayed the onset of LC3 lipidation (defined as the appearance of LC3 on ATG13 or DFCP1 puncta) by twofold (Fig. 3h,i). We concluded that COPI facilitates the progression from the autophagosome nucleation to the elongation step.

### ATG9 vesicles contribute to autophagosome nucleation

**Figure 4:**
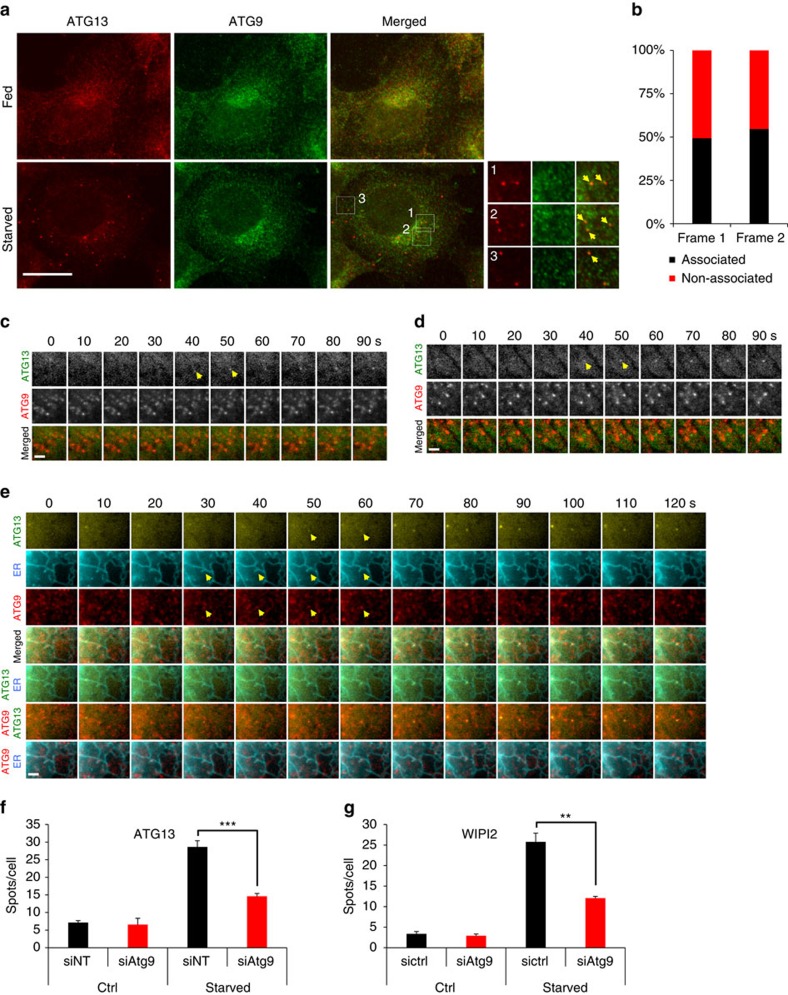
ATG9 promotes the formation of ATG13 puncta. (**a**) HEK293 cells were either fed or starved for 1 h, immunolabelled for ATG13 and ATG9 and imaged by wide-field microscopy. Arrowheads in inserts point at ATG13 particles associating with ATG9. Bar corresponds to 10 μm. (**b**) Values are ATG13 particles in **c**,**d** associating with ATG9 particles in the first two frames from their emergence. From analysis of 75 montages. (**c**,**d**) Wide-field live-cell imaging of starved HEK293 cells stably expressing GFP-ATG13 and mRFP-ATG9. Representative montages of ATG13 particles forming in association with ATG9 (**c**) or not (**d**) are shown. Arrowheads point at the ATG13 particles in the first two frames from their emergence, the same that were used for the analysis in **b**. (**e**) Wide-field live-cell imaging of starved HEK293 cells expressing stably GFP-ATG13 and mRFP-ATG9, and transiently CFP-ER. Representative montage of ATG13 particle forming on a tubular extension of ER previously hosting an ATG9 vesicle is shown. Arrowheads point at the ATG13 particle in the first two frames from its emergence and at the associating extension of ER and ATG9 vesicle. (**f**,**g**) HEK293 cells were transfected with non-targeted (siNT) or ATG9 (siATG9) siRNA, starved for 1 h, immunolabelled for ATG13 or WIPI2 and imaged by confocal laser scanning microscopy. Values are means±s.e.m. puncta of ATG13 (**f**) or WIPI2 (**g**) per cell, for at least five different fields with 15–30 cells each. Significance levels were determined with unpaired *t*-tests. Bar in **c**–**e** corresponds to 300 nm. **P=0.01%; ***P=0.001%.

Our results (Fig. 1) suggest that, although ER–Golgi bi-directional traffic is required for the earliest steps of autophagosome formation, a sizable pool of autophagosomes do not form in any apparent association with ERES. We hypothesized that an alternative candidate source could be ATG9 vesicles. These vesicles are not part of the ER, but have been extensively linked with both ERES and the ULK1 complex. For example, yeast Atg9 binds to the COPII complex and associates with ERES during autophagosome formation^22^,^35^, whereas mammalian ATG9 vesicles associate with the ULK1 complex and with omegasomes^13^,^32^,^44^. We found that endogenous ATG13 almost always associated with endogenous ATG9 (Fig. 4a). In multiple experiments using three different antibodies against ATG9, we found that ∼85% of ATG13 puncta co-localized with ATG9 (Supplementary Fig. 4). However, in live cells only approximately half of the ATG13 particles emerged in clear proximity to ATG9 vesicles (Fig. 4b–d). Importantly, we often observed that ATG9 vesicles localized on a tubular appendix of ER that would subsequently associate with a nascent ATG13 particle (Fig. 4e). Finally, in agreement with a previous study^32^, knock down of ATG9 decreased ATG13 and WIPI2 puncta under autophagy-inducing conditions by ∼50% (Fig. 4f,g), suggesting that ATG9 has a functional role in autophagosome nucleation.

### Multiplex association of ATG13 with ERES-ATG9-VMP1

**Figure 5:**
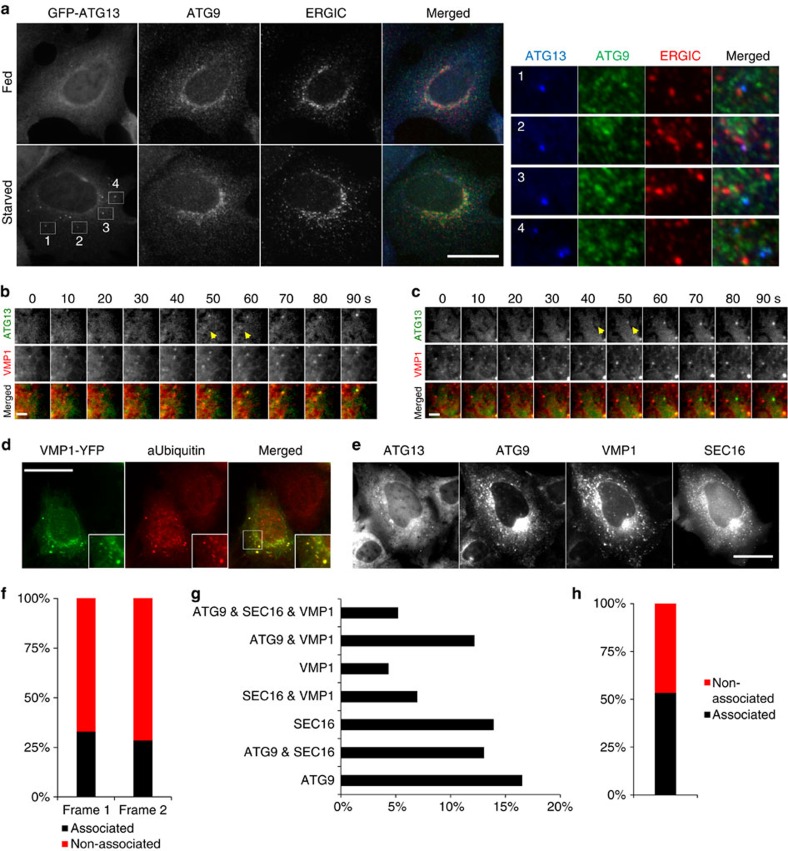
Combination of ERES-ATG9-VMP1 compartments can only partially predict the site of autophagosome nucleation. (**a**) HEK293 cells stably expressing GFP-ATG13 were fed or starved, immunolabelled for ATG9 and ERGIC53 and imaged by wide-field microscopy. Bar corresponds to 10 μm. (**b**,**c**) Wide-field live-cell imaging of starved HEK293 cells expressing stably GFP-ATG13 and transiently VMP1-mCherry. Representative montages of ATG13 particles forming in association with VMP1 (**b**) or not (**c**) are shown. Arrowheads point at the ATG13 particles in the first two frames from their emergence, the same that were used for the analysis in **f**. (**d**) HEK293 cells transiently expressing VMP1-YFP were immunolabelled for ubiquitin and imaged by wide-field microscopy. Representative images are shown. Bar corresponds to 10 μm. (**e**) Wide-field live-cell imaging of starved HEK293 cells expressing stably GFP-ATG13 and mRFP-ATG9 and transiently CFP-SEC16 and VMP1-LSS-mKate2. Representative images of a cell expressing the four proteins are shown. Bar corresponds to 10 μm. (**f**) Values are ATG13 particles in **b**,**c** associating with VMP1 in the first two frames from their emergence. From analysis of 67 montages. (**g**,**h**) Values are ATG13 particles associating with any of ATG9, SEC16 and VMP1 (**g**) or with different combinations of them (**h**) in the first two frames from their emergence. Bar in **b**,**c** corresponds to 300 nm.

We tested if puncta combining ATG9 and ERGIC would associate with all ATG13 particles. In fixed cells, the majority of ATG13 particles (over 85%) associated only with ATG9 (Fig. 5a, inset 1, 3 and 4), and a minority (<15%) with only ERGIC or with both (Fig. 5a; inset 2 purple particle where ATG9 and ERGIC co-localize). Under these conditions, very few ATG13 particles did not co-localize with either ATG9 or ERGIC.

We explored if a third autophagy-related protein that forms puncta may contribute to autophagosome nucleation. We thought that VMP1, a transmembrane ER protein implicated in the early steps of autophagosome formation^44^,^45^,^46^, may play this role. Though approximately one third of the ATG13 particles emerged in close proximity to VMP1 (Fig. 5b,c,f), knock down of VMP1 did not affect the number of puncta formed by ATG13 or WIPI2 under autophagy-inducing conditions (Supplementary Fig. 5), suggesting that it is not functionally involved in the nucleation step. Intriguingly, almost all puncta of the exogenous VMP1 co-localized with ubiquitin (Fig. 5d), suggesting that VMP1 may be ubiquitinated or trigger the ubiquitylation of another protein.

In the live imaging experiments, the sum of emerging ATG13 particles that associate with ERES/ERGIC (∼35%), ATG9 (∼55%) and VMP1 (∼30%) suggested that the three compartments combined may provide the platform to nucleate all autophagosomes. We tested this by four-colour live-cell imaging in cells expressing GFP-ATG13, mRFP-ATG9, CFP-SEC16 and VMP1-LSSmKate2 (Fig. 5e). Emerging ATG13 particles associated more often with ATG9 compared with ERES or VMP1 (Fig. 5g), but only 55% of them associated with any of these compartments (Fig. 5h). We hypothesized that the size of the early ATG13-positive ULK1 structures and the other compartments may make it difficult to accurately evaluate their association by conventional imaging and we turned to protocols of super resolution to further address this question.

### Distribution of ATG13 on autophagosomes at super resolution

**Figure 6:**
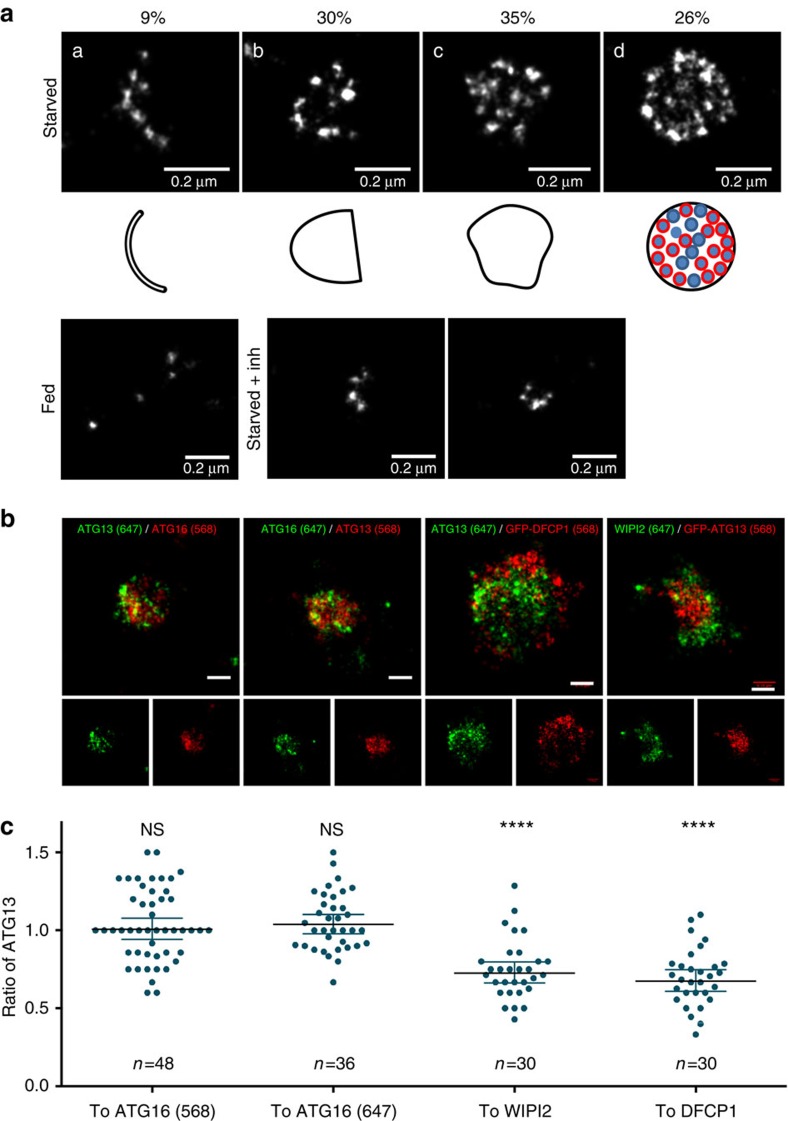
ATG13 shows unique distribution pattern on autophagosome membranes. (**a**) HEK293 cells were fed or starved in the presence or absence of VPS34 inhibitor for 1 h, immunolabelled for endogenous ATG13 and imaged by dSTORM. The structures of observed ATG13 particles under starved conditions were assigned to four different patterns: crescent shaped (a), semi-spherical (b), quasi-spherical (c) and spherical (d). Representative examples of reconstructed super-resolution images are shown for each pattern. Shapes below the images describe the outline of each observed pattern. Red circles within the spherical pattern correspond to areas of higher density of identified molecules. Values are the percentage of ATG13 particles corresponding to each pattern; from 120 particles analysed. (**b**) HEK293 cells stably expressing GFP-DFCP1 or GFP-ATG13, or the parental cell line were starved, immunolabelled for ATG13, WIPI2, ATG16 or GFP and imaged by dSTORM. Representative examples of reconstructed super-resolution images are shown. Bar corresponds to 0.15 μm. (**c**) The area occupied by ATG13 and ATG16 (labelled with Alexa Fluor 647- or CF 568- conjugated secondary antibody), WIPI2 or GFP-DFCP1 in the reconstructed super-resolution images in **b** was quantitated. Values are the ratios of ATG13–ATG16, WIPI2 and DFCP1 in each of the analysed particles. Significance levels were determined with one-sample *t*-test against a theoretical mean of 1 (if ATG13 and ATG16, WIPI2 or DFCP1 occupied the same area), with Bonferroni correction for multiple comparisons. ****P=0.0001%.

Mature mammalian autophagosomes are 0.5–1.5 μm in diameter^47^, thus conventional light microscopy (maximum lateral resolution of 250 nm) may not be able to resolve the autophagosome precursors. This may also complicate the efforts to identify the cellular organelles comprising the membrane source for autophagosome nucleation^48^. We developed a protocol for dSTORM to observe the autophagosome precursors and how they associate with the ATG9 and ERGIC compartments at super resolution for the first time. In starved cells, the ATG13 puncta corresponded to pleiomorphic structures no more than 300 nm in diameter, with ATG13 distributed on a granular pattern, as if it resided on distinct vesicles or focal points on a flat membrane (Fig. 6a). The ATG13 dots were further organized in four distinct patterns: crescent shaped (a), semi-spherical (b), quasi-spherical (c) and spherical. They showed a bias towards the outside of the cluster, often organizing in a ring-like structure (more obvious in the spherical pattern). The different distribution patterns may correspond to the two-dimensional projections of the same three-dimensional cup-shaped pattern when observed from different angles (ranging from spherical when observed from the top to crescent shaped when observed from the side). Alternatively, the distribution of ATG13 may change during progression from the nucleation to the elongation step of autophagosome formation. On the other hand, in fed cells ATG13 seldom showed these patterns residing mainly in small single puncta (Fig. 6a). We also starved cells in the presence of a highly selective VPS34 inhibitor^49^ to examine the morphology of the earliest autophagosome precursors before PI3P is formed^16^. This inhibitor allows the formation of puncta by ATG13 (Supplementary Fig. 6a,c,d) or FIP200 (another member of the ULK1 complex, Supplementary Fig. 6a,b), abolishes the formation of puncta by the PI3P-binding protein WIPI2 (Supplementary Fig. 6b) and by the component of the lipidation machinery ATG16 (Supplementary Fig. 6c), but allows the formation of smaller puncta by the ULK1 complex that co-localize with ATG9 vesicles (Supplementary Fig. 6d). The ATG13 puncta forming under inhibition of PI3P synthesis comprised clusters of structures smaller than 100 nm in diameter (Fig. 6a), which in some cases organized in a crescent-shaped pattern, but never extended to larger spherical patterns. We believe that these structures represent the earliest autophagy-specific assemblies observed here at super resolution for the first time.

We also used two-colour dSTORM to evaluate the association of ATG13 with the downstream autophagic machinery at super resolution. We first examined the relationship of ATG13 with ATG16, which coincides with the site of LC3 lipidation (isolation membrane)^50^. We found that ATG16 and ATG13 were confined on the same space and adopted a similar distribution pattern (Fig. 6b,c). We have also examined the relationship of ATG13 with the PI3P-rich compartment, as represented by WIPI2 and DFCP1. ATG13 occupied a smaller area compared with the PI3P-binding proteins and often was more dense inwards of the ring formed by DFCP1 and WIPI2 (Fig. 6b,c).

### Relationship of the ULK1 complex with ATG9 and ERES/ERGIC

**Figure 7:**
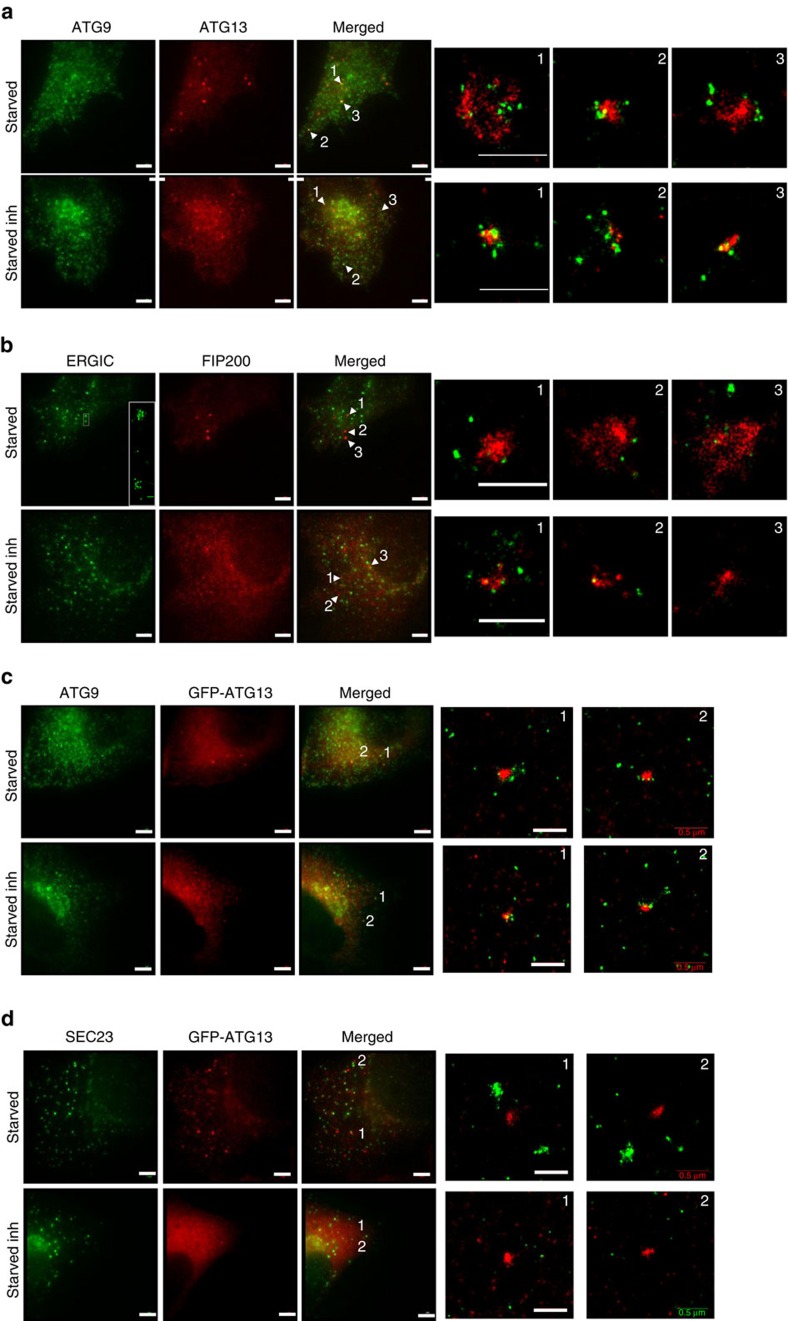
Autophagosomes associate with ATG9 and ERGIC membrane compartments. (**a**,**b**) HEK293 cells were starved in the presence or absence of VPS34 inhibitor for 1 h, immunolabelled for ATG13 and ATG9 (**a**) or FIP200 and ERGIC53 (**b**), and imaged by dSTORM. (**c**,**d**) HEK293 cells stably expressing GFP-ATG13 were starved in the presence or absence of Vps34 inhibitor for 1 h, immunolabelled for ATG13 and ATG9 (**c**) or SEC23 (**d**), and imaged by dSTORM. Conventional images and super-resolution magnifications are shown. Scale bars in wide-field images: 5 μm. Scale bars in super-resolution images, 0.5 μm.

**Figure 8:**
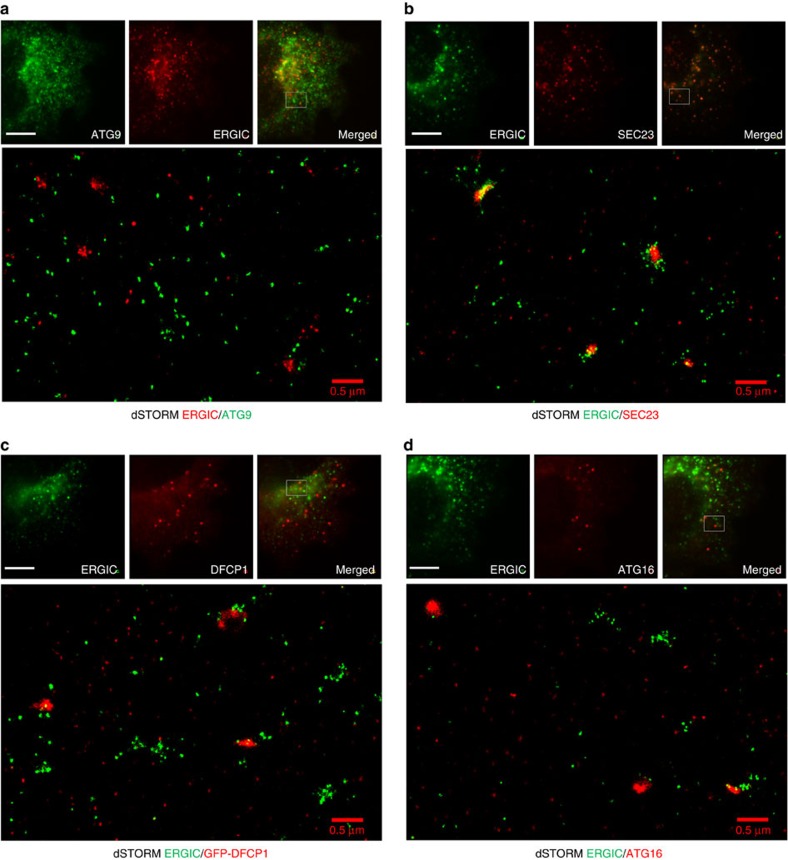
dSTORM imaging of ERGIC. Parental HEK293 cells (**a**,**b**,**d**) or HEK293 cells stably expressing GFP-DFCP1 (**c**) were starved, immunolabelled for ERGIC53 and ATG9 (**a**), SEC23 (**b**), GFP(**c**) or ATG16 (**d**) and imaged by dSTORM. Representative conventional images and super-resolution magnifications are shown. Scale bars in wide-field images, 5 μm. Scale bars in super-resolution images, 0.5 μm.

ATG9 and ERES/ERGIC compartments comprise clusters of vesicles of 20–30 and 60–70 nm in diameter, respectively^14^,^25^, which cannot be resolved by conventional light microscopy. We used two-colour dSTORM to re-evaluate the association of the ULK1 complex with the ATG9 and ERES/ERGIC compartments. ATG9 localized on vesicles <50 nm in diameter, dispersed in the cytosol and often organized in clusters (Fig. 7a,c). Conversely, ERGIC53 localized on small vesicles primarily clustered next to ERES, with some also dispersed in the cytosol (Fig. 7b). Under autophagy-inducing conditions multiple ATG9 vesicles surrounded the ATG13 structures independent of PI3P synthesis (Fig. 7a,c), suggesting that ATG9 contributes to autophagosome nucleation. Moreover, scattered ERGIC vesicles (but not large clusters) surrounded FIP200 structures independent of PI3P synthesis, although less often compared with ATG9 vesicles (Fig. 7b), suggesting that ERGIC also contributes to autophagosome nucleation. FIP200 was used instead of ATG13 due to antibody constraints. In contrast, ATG13 did not associate with the ERES marker SEC23 (Fig. 7d). Of note, ATG9 and ERGIC did not co-localize in dSTORM even when they appeared to be close in wide field (Fig. 8a). In contrast, SEC23 and ERGIC, which co-localized in wide-field images, were also closely associating in super-resolution images (Fig. 8b). These data suggest that regions of extensive overlap between ERES and ERGIC (Fig. 8b), which presumably constitute the canonical ERES/ERGIC compartment, are not in direct association with the site of autophagosome biogenesis. Moreover, in contrast to recent reports^23^,^24^, DFCP1 (a PI3P-binding protein) and ATG16 (part of the LC3 lipidation machinery) also co-localized only with scattered ERGIC vesicles (Fig. 8c,d) and not with the canonical ERGIC compartment. Our super-resolution imaging data suggest that autophagy-specific structures (marked by ATG13, ATG16, DFCP1, FIP200 and ATG9) are in very close proximity with each other, but not with pre-existing compartments such as the ERES and ERGIC.

### ATG13 targets a tubulovesicular compartment adjacent to ER

**Figure 9:**
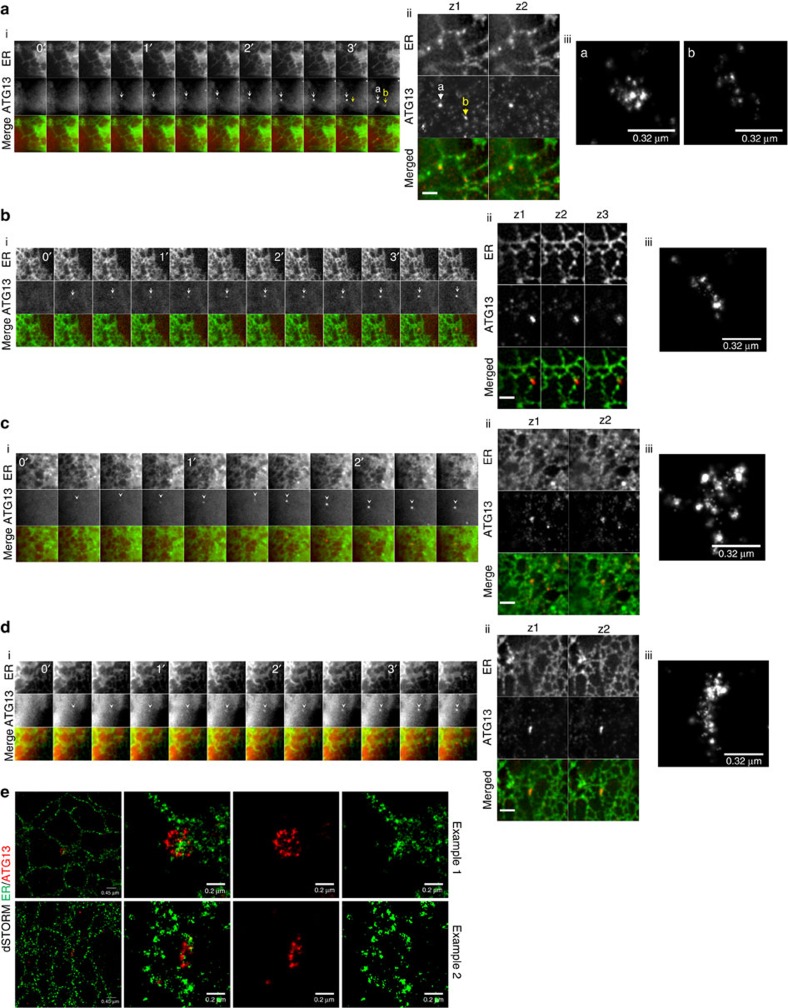
ATG13 targets the ER. (**a**–**d**) HEK293 cells expressing stably GFP-ATG13 and transiently mCherry-dgk1 (ER marker) were starved, live-imaged by wide-field microscopy, fixed on stage and immunolabelled for ATG13 (secondary antibody conjugated to Alexa Fluor 647). The cells were relocated and imaged by SIM (for mCherry and Alexa Fluor 647) and dSTORM (for Alexa Fluor 647). Montages of representative ATG13 particle formation events from the live-cell imaging step (i), different *z* stacks from the SIM step (ii) and blow-ups of the ATG13 particles from the super-resolution dSTORM images (iii) are shown. Each figure (**a**–**d**) corresponds to an independent example. (**e**) HEK293 cells expressing transiently GFP-dgk1 (ER marker) were starved, immunolabelled for GFP and imaged by dSTORM. Two representative examples are shown. Bars in SIM images correspond to 1 μm.

We have previously used live-cell imaging to show that the early ATG13 assemblies associate with the ER^16^, but we could not resolve if they target ER or an adjacent compartment. We proceeded to answer this question combining live-cell imaging with super-resolution microscopy. After imaging GFP-ATG13 and mCherry-ER in live cells, the samples were fixed on stage to halt the progress of autophagosome formation. This allowed us to identify ATG13 particles whose provenance was known. The samples were then immunolabelled for ATG13 and subjected to super-resolution microscopy: first structured illumination microscopy (SIM) of both ATG13 and ER, and then dSTORM of ATG13. Examples of four particles representative of our findings are shown in Fig. 9a–d. Both early particles (particle b with lifetime of 40 s in Fig. 9aii) and late particles (particle a in Fig. 9aii and particles in Fig. 9b–dii) were found to associate with ER in three different modes: either on a natural extension of ER, but devoid of the ER marker (Fig. 9aii particles a and b, and Fig. 9bii), partially on a natural extension and partially overlapping with ER (Fig. 9cii), or completely overlapping with ER (Fig. 9dii). To verify this consistent pattern of ER association, we examined localization of endogenous ATG13 on ER by dSTORM. Although here we lacked the information on lifetime, in agreement with the SIM data above, ATG13 particles were found assembled on an ER extension (Fig. 9e, example 1) or overlapping with the ER strand (Fig. 9e, example 2). Remarkably, the characteristic spherical pattern with a ring-like morphology (see Fig. 6a, last structure on top) was seen to surround the ER extension (Fig. 9e, example 1), consistent with the idea that this is the critical intermediate in the pathway.

**Figure 10:**
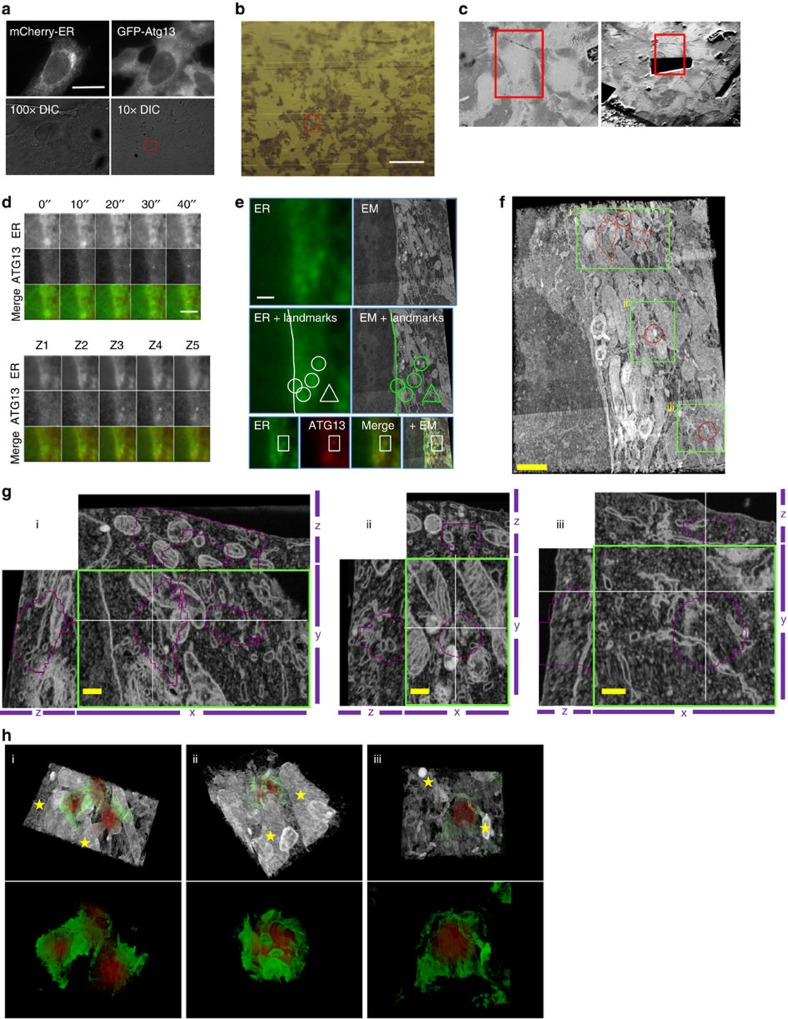
Correlative light and electron microscopy of ATG13 and ER. HEK293 cells stably expressing GFP-ATG13 and transiently expressing mCherry-dgk1 (ER marker) were starved, subjected to live-cell imaging by wide-field microscopy and fixed on stage. (**a**) Fluorescent images of the frame capture just before the fixation, × 100 and × 10 DIC images of the fixed cells are shown. Red box in × 10 DIC image indicates the cell of interest. (**b**) Image of the resin-embedded sample. Cell of interest located in red box. (**c**) Resin blocks were trimmed down to a block face of 1 mm^2^ and mounted on stub for imaging in an Auriga focused ion beam scanning electron microscopy (FIB-SEM, Carl Zeiss). Overview images before (left) and after milling (right) indicating the cell of interest with a red box. (**d**) Montage of an ATG13 particle formation from the live-cell imaging step and *z* stacks after fixation (particle ii in **f**). (**e**) Overlays of light and electron microscopy images. Light and electron microscopy images were correlated using landmarks identified in both (shown in white and green lines, circles and triangles). (**f**) Three-dimensional (3D) opacity rendering of the FIB-SEM image stack. The areas outlined in red within the green boxes indicate ATG13 particles. Particle ii is the one that could be traced throughout the experiment and was identified in both live-cell and FIB-SEM imaging. ATG13 Particles in boxes i and iii could be identified from the wide-field and fluorescence image, but their provenance by live imaging could not because they were on a different focal plane from particle ii. (**g**) Magnification of the area within the green boxes in **f** (i–iii). Shown are the *XY* view from the middle of the ATG13 signal, and orthogonal *XZ* and *YZ* views along the thin white lines. (**h**) 3D Opacity rendering of the cropped FIB-SEM stacks in **g** with overlay of the ATG13 signal (red). Rendered in green are the membranes detected in the FIB-SEM stack that are in proximity of the ATG13 particle. Stars indicate mitochondrial membranes. Bars: 10 μm (**a**), 50 μm (**b**), 5 μm (**d**–**e**), 1 μm (**f**) and 0.25 μm (**g**).

To visualize potential membranes that may be involved in the nucleation of ATG13 structures, we combined light microscopy with focused ion beam scanning electron microscopy (FIB-SEM). We identified a total of five particles for which we could correlate the light and electron microscopy images (Fig. 10f, red outlines in boxes i–iii). For one of the particles (Fig. 10f, particle ii) live-cell imaging also allowed us to derive its exact lifespan (40 s; Fig. 10a–e). In all cases, the light microscopy signal of ATG13 correlated with a pleiomorphic tubular–vesicular compartment (Fig. 10g,h) and, as expected, there were no classical autophagosome-like complete structures visible (that is, double-membrane vesicles). This tubulovesicular compartment was for the most part cradling the ATG13 region (Fig. 10h; note red signal surrounded by green membranous elements) and at the same time contained membrane elements extending within the space of the ATG13 structure (Fig. 10g; note membranes within the purple outlines). This organization is compatible with the organization of the ATG9/ERGIC structures that surrounded the ATG13 particles observed by dSTORM in Fig. 7. Mitochondrial membranes (stars in Fig. 10h) were also nearby. The tubulovesicular organization of the membranes underlying the ATG13 signal was similar in all cases, suggesting that it is representative of the morphology of these structures.

## Discussion

We have tried to identify where the ULK1 complex nucleates autophagosomes using as main surrogate the component of the complex ATG13. We show that both ERES/ERGIC and ATG9 compartments are functionally contributing to autophagosome nucleation, but ATG9 associates more ubiquitously with the early ULK1 structures. We have used super-resolution microscopy to capture the morphology of the ATG13 compartment and its relationship with the autophagic machinery and the ERES/ERGIC and ATG9 compartments. We show for the first time at super resolution that the ATG13 compartment associates with clusters of ATG9 vesicles or with isolated elements of ERGIC, which cannot be resolved by conventional light microscopy, but rarely with ERES. Moreover, combining light with electron microscopy we correlate the ATG13 early punctum with a tubulovesicular membrane compartment surrounded by ER and mitochondria. This compartment is compatible with the tubulovesicular ATG9 compartment previously described^14^,^32^,^51^,^52^. Finally, building up on our previous study^16^, we combine several imaging modes (dual-colour SIM and dSTORM and CLEM) to describe the association of the ATG13 structures with the ER at sub-diffraction resolution. Combining everything we suggest that the nucleation of autophagosomes occurs in regions, where the ULK1 complex coalesces with ER and the ATG9 compartment.

We have shown that the associating ULK1 and ATG9 structures previously reported^13^,^32^,^53^ comprise multiple ATG9 vesicles <50 nm in diameter swarming around the ATG13 puncta (Fig. 7a,c). These structures may also correspond to the tubulovesicular compartment observed by electron microscopy (Fig. 10g,h). The association between ATG9 and early ATG13 structures is independent of PI3P synthesis (Fig. 7a,c), as previously reported^54^. Under these conditions, where no isolation membrane is evident, the ATG13 assemblies already begin to assume quasi-spherical morphology (Fig. 6a; starved+inh). In our opinion, this is strong evidence that some of the membrane organization function necessary for autophagosome formation exists within the ULK1 complex. Given the capacity of yeast ULK1 to bind highly curved vesicles similar with ATG9 vesicles^55^ and the phosphorylation cascades underpinning activation of the complex during autophagy^56^, this arrangement is possibly self-propelled. We speculate that this type of association between ATG9 and the early ATG13 structures may suggest that the ULK1 complex usurps membrane from the ATG9 compartment to nucleate a new autophagosome, while ATG9 only transiently incorporates to the autophagosome membrane as it was previously shown^53^. Intriguingly, inactivation of the yeast ULK1 complex causes the tubulovesicular ATG9 compartment to resolve into distinct vesicles^14^. A layered network of interactions that may bring the yeast ULK1 complex in gradually closer proximity to the ATG9 compartment was also recently suggested^57^.

The ULK1 structures also adjoin ER and overlap with scattered ERGIC-derived vesicles, but not with the canonical ERES/ERGIC vesicular clusters (Figs 7 and 8), even if their assembly is facilitated by the functional integrity of ERES (Fig. 1). Why is the ER-to-Golgi traffic required for ATG13 puncta formation, but these puncta do not localize on canonical ERES? One possibility is that the canonical ERES/ERGIC may affect the ATG13 indirectly. Cell-free assays have recently shown that ERGIC membranes recruit the component of the VPS34 complex ATG14 and, in PI3P- and COPII-dependent manner, generate the membrane template for LC3 lipidation^23^,^24^. Therefore, ERGIC may affect the ATG13 indirectly through activation of the VPS34 complex. The existence of a positive feedback loop from the VPS34 complex that reinforces the translocation of ATG13 to its early structures is compatible with this hypothesis^16^.

The yeast autophagic machinery occupies distinct sites on forming autophagosomes^22^,^35^. Using super-resolution microscopy of endogenous autophagy proteins, we here provide a spatial context of the physical and functional interactions among the autophagic machinery in mammalian cells. We can distinguish at least two layers of components: a layer at the autophagosome periphery comprising the PI3P effectors (and potentially the VPS34 complex), and an inner layer comprising the ULK1 complex (ATG13 and FIP200) and the lipidation machinery (ATG16; Fig. 6). This arrangement should allow ATG16 to interact with WIPI2 and FIP200 (refs 58, 59) and LC3 to accumulate within the DFCP1 ring^8^,^16^. In agreement with this spatial arrangement, disruption of ATG16 binding to FIP200 inhibits autophagy^60^. Previous studies have suggested that SNARE-mediated fusion of vesicles is involved in autophagosome expansion^11^,^51^. We speculate that if the elongation of the isolation membrane occurs through vesicle fusion at its extremities, this arrangement is compatible with membrane handover from the PI3P-rich area to the area hosting the ULK1 complex and the lipidation machinery.

Our aim was to understand the provenance and mode of formation of the early ULK1 structure to identify where autophagosomes are nucleated. On the basis of our findings, we suggest that upon mTOR inactivation and the subsequent phosphorylation/dephosphorylation cascades driven by alterations in the ULK1 kinase activity, the ULK1 complex clusters on tubulovesicular elements made up of ER tubules and ATG9 vesicles, with some contribution from ERGIC/ERES vesicles. We suggest that this structure, which can be thought of as an autophagy-specific ERES and already takes on a quasi-spherical shape, is the origin of autophagosomes. Subsequently, the VPS34 complex would generate PI3P enriched membranes that enable the original structure to expand and to attract the lipidation machinery. At the same time, traffic from the nearby ERGIC and ERES would ensure additional supply of membranes and the translocation and activation of the lipidation machinery. At this stage, we imagine that the growing phagophore could functionally interact with additional membrane compartments (mitochondria, endosomes, plasma membrane and so on) for expansion and maturation, as recently shown by advanced immunolabelling and electron microscopy techniques^61^.

## Methods

All chemicals were obtained from Sigma-Aldrich unless otherwise stated. FLI06 was obtained from Tocris Bioscience. VPS34 inhibitor VPS34-IN1 was a kind gift of I.J. Ganley (MRC Protein Phosphorylation and Ubiquitylation Unit, College of Life Sciences, University of Dundee, Dundee, UK).

### Antibodies

The antibodies used in the course of this work were: mouse anti-ATG13 (Millipore, cat. no. MABC46; immuno-fluorescence (IF) 1:100), rabbit anti-ATG101 (Sigma, cat. no. SAB4200175; IF 1:100), mouse anti-WIPI2 (AbD Serotec, cat. no. MCA5780GA; IF 1:200), rabbit anti-ATG16 (MBL, cat. no. PM040; IF 1:100), mouse anti-GFP (Roche, cat. no. 11814460001; IF 1:100), rabbit anti-GFP (gift of L. Roderick, Department of Cardiovascular Sciences, KU Leuven), goat anti-SEC23 (Santa Cruz, cat. no. sc-12107; IF 1:100), rabbit anti-SEC23IP (Sigma, cat. no. HPA038403; IF 1:100), rabbit and mouse anti-ERGIC53 (Santa Cruz, SC66880 and SC271517; IF 1:100), mouse anti-multi ubiquitin (Cayman Chemicals, cat. no. 14220; IF 1:100), mouse anti-GAPDH (Biogenesis, cat. no. 4699-955; WB 1:10000), rabbit anti-LC3 (Sigma, cat. no L7543; WB 1:2000), rabbit anti-ARCN1 (Sigma, cat. no. HPA037573; WB 1:1000), rabbit anti-COPB2 (Novus Biologicals, cat. no. NB120-2899; WB 1:1000) mouse anti-beta COP (a gift from the late Thomas Kreiss), hamster anti-ATG9^62^ (a gift of S. Tooze, The Francis Crick Institute, London), rabbit anti-ATG9 (Cell Signalling, cat. no. 13509; IF 1:100), rabbit anti-ATG9 (GeneTex, cat. no. GTX128427; IF 1:100), goat anti-mouse fluorescein isothiocyanate (FITC) (Jackson ImmunoResearch, cat. no. 115-095-062; 1:100), goat anti-rabbit FITC (Jackson ImmunoResearch, cat. no. 111-085-045; 1:100), goat anti-mouse tetramethylrhodamine TRITC (Jackson ImmunoResearch, cat. no. 115-025-062; 1:100), goat anti-rabbit TRITC (Jackson ImmunoResearch, cat. no. 115-025-144; 1:100), goat anti-mouse Alexa Fluor 647 (Molecular Probes, A-21236; 1:500-1:1000), goat anti-rabbit Alexa Fluor 647 (Molecular Probes, A-21244; 1:500-1:1000), CF568 goat anti-mouse IgG (Biotium, cat. no. 20301; 1:100) and CF568 goat anti-rabbit IgG (Biotium, cat. no. 20099; 1:100).

### Plasmids

The plasmid for the expression of mCherry-SEC16 was a gift of J. Nunnari (Department of Molecular and Cellular Biology, University of California, Davis). To obtain pECFP-C1-SEC16, the SEC16 open reading frame (ORF) was cleaved with BglII/SalI from the plasmid expressing mCherry-SEC16 and was subcloned in the pECFP-C1 vector (Clontech). The plasmid for the expression of mRFP-Atg9 was a gift from S. Tooze (London Research Institute, London, UK). The plasmids for the expression of VMP1-GFP and VMP1-YFP were gifts of M. Vaccaro (Institute for Biochemistry and Molecular Medicine, Department of Pathophysiology, School of Pharmacy and Biochemistry, University of Buenos Aires, Buenos Aires, Argentina). To obtain pmCherry-N1-VMP1, the ORF of VMP1 was cleaved from pEGFP-N1-VMP1 by HindIII/BamHI and was subcloned to pmCherry-N1 (Clontech). To obtain pECFP-N1-VMP1, CFP was cleaved from pECFP-N1 (Clontech) by AgeI/BsrGI and was subcloned in the pEGFP-N1-VMP1 vector from which the ORF of GFP was cleaved by AgeI/BsrGI. To obtain pLSSmKate2-N1-VMP1, the ORF of VMP1 was cleaved from pECFP-N1-VMP1 by SacI/BamHI and was subcloned to the pLSSmKate2-N1 vector that was purchased from Addgene^63^.

### Cell culture and generation of cell lines

Stable GFP-ATG13 cell line was generated and characterized in a previous study^16^. Stable GFP-ATG13- and mRFP-ATG9-expressing cell lines were selected in 800 μg ml^−1^ geneticin and 100 μg ml^−1^ zeocin. Transfections of HEK293 cells were performed using X-treme GENE 9 DNA transfection reagent (Roche Applied Science) according to the manufacturer's instructions. Short interfering RNAs (siRNAs) for ATG9, VMP1, δCOP, β'COP and non-targeted were ON-TARGETplus siRNAs from Dharmacon.

### Western blots

Un-cropped versions of the western blots in Fig. 2 are shown in Supplementary Fig. 7.

### Starvation of HEK293 cells for induction of autophagy

Unless stated otherwise, for amino-acid starvation, cells were washed three times with pre-warmed starvation medium (140 mM NaCl, 1 mM CaCl_2_, 1 mM MgCl_2_, 5 mM glucose, 1% BSA and 20 mM HEPES pH 7.4), followed by incubation in starvation medium for 1 h.

### IF microscopy

Staining for IF microscopy was done as described previously^64^. In brief, cells seeded on glass coverslips were fixed with 3.7% formaldehyde, permeabilised with NETgel (150 mM NaCl, 5 mM EDTA, 50 mM Tris·Cl, pH 7.4, 0.05% (v/v) NP-40, 0.25% (w/v), gelatin (from bovine skin; Sigma, cat. no. G-6650) and 0.02% (w/v) sodium azide) containing 0.25% NP40, and stained for 30 min with primary Ab (at the dilutions described above). Cells were washed for 5 min with NETgel (three times), stained for 30 min with secondary Ab (at the concentrations described above), and mounted with Aqua Poly Mount mounting medium (Polysciences, cat. no. 18606). All steps were performed at room temperature. Staining for dSTORM imaging was done in the same way, but instead of mounting, cells were washed seven times with PBS and were stored in PBS at 4 °C.

### Confocal imaging

Images for quantitation of puncta were captured with an Olympus FV1000 confocal microscope using a × 60 1.4 numerical aperture (NA) objective (Olympus). The images were captured from randomly selected fields of view.

### Quantitation of puncta

Quantitation of puncta was performed using the Imaris software from Bitplane/Andor.

### Epi-fluorescence imaging

Images of fixed cells were acquired using a Zeiss Axio Imager D2 wide-field epi-fluorescence microscope equipped with a × 63 1.4 NA lens, AxioCam HR CCD camera (Zeiss) and HXP 120C metal halide light source (Zeiss).

### Live-cell imaging

Live-cell imaging was performed as previously described^64^,^65^. In brief, two wide-field imaging systems were used to capture images of live cells: a CellR imaging system (Olympus) and a Nikon Ti-E-based system. Cells were plated onto 60 mm dishes and transiently transfected with the relevant plasmids. The next day the transfected cells were transferred onto 22 mm diameter glass coverslips (BDH) and on the third day the individual coverslips were secured in an imaging chamber with 2 ml of cell medium or starvation medium added as indicated. The assembled imaging chamber was secured onto the microscope stage, and cells were maintained at 37 °C using a Solent Scientific full enclosure incubation system (used with the Olympus CellR), or an OKO Labs full enclosure incubation system (used with the Nikon Ti-E). The CellR imaging system was equipped with a × 100 1.4 NA objective (Olympus), Polychrome V monochromator (TILL Photonics) and a Hamamatsu Orca CCD camera. CFP, GFP, and mRFP were excited at 425, 488 and 585 nm, respectively, typically using 15 nm bandwidth and 20% transmission. Band-pass filters at 467–500, 505–545 and 605–680 nm were used to collect fluorescent light from CFP, GFP and mRFP, respectively. The Nikon Ti-E-based system comprised a Nikon Ti-E microscope, × 100 1.4 NA objective (Nikon), SpecraX LED illuminator (Lumencor), 410/504/582/669-Di01 and Di01-R442/514/561 dichroic mirrors (Semrock), Hamamatsu Flash 4.0 sCMOS camera, emission filter wheel (Sutter Instruments) and was controlled using Nikon Elements software. Excitation and emission filters (all from Semrock) were as follows: CFP 434/17 (ex) 480/17 (em), GFP 488/10 (ex) 525/30 (em), mRFP 560/25 (ex) 607/36 (em) and LSSmKate 488/10 (ex) 607/36 (em). LSSmKate2 has a large Stokes shift (excitation/emission maxima at 460/605 nm), absorbing in the green region and emitting in the red region, and as a result it provided an additional red colour to be used in parallel with mCherry^63^.

### Analysis of live-cell imaging videos

Live-cell imaging videos were analysed with Fiji (Fiji Is Just ImageJ) Open Source software as previously described^64^,^65^. In brief, at first montages of independent events were created from the captured videos. We define as an independent event all the frames that correspond to the formation and collapse of one GFP-ATG13 particle, starting and finishing with the frames in which the fluorescence of the GFP-Atg13 particle is emerging clearly above the fluorescence of the cytosolic GFP-ATG13 (for example, Fig. 1c). Frames corresponding to time points before the beginning or after the end of a particular event were carefully scanned, and events corresponding to particles moving out of focus and then back in were excluded from the analysis. For the analysis of the distance between GFP-ATG13 and the different markers, the same coordinates were used to create montages of the other markers and composite images of the two channels were created to analyse the co-localization of the two proteins. Lines were drawn crossing the sites of closest association (judged by eye in large magnification) and line plots of the fluorescence intensity were used to decide on association. Association was scored when the same pixels had signal above the background for both channels.

### dSTORM imaging

Samples for dSTORM were labelled with goat anti-rabbit and goat anti-mouse secondary antibodies conjugated to Alexa Fluor 647 (Thermo Fisher Scientific) or CF 568 (Biotium), a novel super-resolution dye that is compatible with Alexa Fluor 647 (ref. 66). Secondary antibodies conjugated to Alexa Fluor 647 were used for single labelling experiments, while antibodies conjugated to CF 568 (Biotium) were additionally used for double labelling experiments. Labelled samples were mounted into custom imaging rings and immersed in 500 μl dSTORM imaging buffer comprising (all from Sigma) catalase (1 μg ml^−1^), TCEP (0.8 μM, glucose (40 mg ml^−1^), glucose oxidase (50 μg ml^−1^), glycerine (12.5%), KCl (1.25 mM), Tris-HCl (1 mM) and MEA-HCl (100 nM)). The buffer was overlaid with a thin layer of mineral oil to reduce gas exchange and the imaging ring assembly was fixed onto the stage of the microscope. dSTORM images were captured using a Nikon N-STORM imaging system, comprising Nikon Ti-E microscope, Nikon 1.49 NA × 100 oil immersion objective, Andor iXon 897 EM-CCD camera, MBP Communications 643 nm laser (300 mW), Coherent Sapphire 561 nm laser (150 mW), Coherent Cube 405 nm laser (100 mW) and Semrock LF405/488/561/635-A-000 multiband filter set. The imaging system was controlled using Nikon Elements software. Laser power (max) measured at the sample was (in mW) 4.1, 27.3 and 65.3 for the 405, 561 and 643 nm lasers, respectively. Images 256 × 256 pixels (0.16 μm per px) were captured at a frame rate of 55 fps with the camera conversion gain set to 2.4 and the electron microscopy gain typically set at 100. At least 15,000 frames were captured per field of view, with the laser illumination switching alternately for dual-labelling experiments. Laser power of 405 nm was ramped during the image capture to maintain blinking frequency as determined by the Nikon software. dSTORM images were reconstructed using Nikon Elements software, starting the analysis from a frame where only isolated blinks were observed across the whole field of view (no residual continuous signal could be seen). Identification of molecules was typically done using default blink identification parameters within the Nikon software, using Minimum Heigth threshold of 750. Chromatic alignment of dSTORM images from dual-labelled samples was ensured by calibrating the system using a slide with 100 nmTetraspeck beads (Life Technologies) and following the chromatic calibration procedure in the Nikon software. Channel crosstalk was excluded by running control experiments, in which the samples were labelled with secondary antibody only for the one channel and images were captured in the other channel. The resolution of our system was determined using dSTORM images of test samples (microtubules) under imaging conditions similar to those described here, in which we have been able to resolve objects separated by a distance of <30 nm. For the regions of interest, shown localization precision of the identified molecules, as estimated by the N-STORM module of the Nikon software^67^, were typically 5–9 nm (CF568) and 3–10 nm (AF647). Identified molecules are displayed as a Gaussian spot of diameter 5 nm with an intensity profile proportionate to the localization precision (spots with a higher precision of localization appear brighter). To filter out identified molecules that did not correspond to the puncta observed by conventional light microscopy, the density was adjusted to 50–60 counts per 100 nm.

### dSTORM topology analysis

The structures of the particles were analysed using the ‘Auto Detect ROI' tool of the Nikon Elements software to draw the outline around the area occupied by each protein. The values of the size of the selected area for each protein of interest were then exported, and the area occupied by ATG13 was divided by the area occupied by each of the other proteins (ATG16, WIPI2 or DFCP1).

### Correlative light and super-resolution microscopy

Cells were seeded on glass bottom gridded dishes (MatTek) and live-cell imaging was performed as described above. Cells were fixed on stage by adding 3.7% formaldehyde at 1:1 ratio to culture medium and a differential interference contrast (DIC) image was captured with a × 10 0.3 NA lens to mark the position of the cells imaged on the grid. After appropriate permeabilisation and immunolabelling for ATG13, coverslips were mounted into custom imaging rings and immersed in 500 μl STORM imaging buffer, transferred to a Nikon N-SIM/N-STORM dual super-resolution microscope (Nikon Ti-E microscope with SIM/STORM adaptations, Andor iXon 897 EM-CCD camera and Nikon 1.49 NA oil immersion objective, with the imaging system controlled using Nikon Elements software) and cells were relocated and imaged first using SIM and thenSTORM. Raw 3D-SIM images were acquired (15 images representing 5 phases and 3 rotations at each focal plane) typically using 100 msec exposure, 5.1 conversion gain and 150 electron microscopy gain and reconstructed into super resolved images using default parameters in the Nikon software. mCherry was excited using a 561 nm laser with emission collected at 607/36; Alexa Fluor 647 was excited using a 643 nm laser with the emission collected at 692/40.

### Correlative light and electron microscopy

Cells were seeded on glass bottom gridded dishes (MatTek) and live-cell imaging was performed as described above. Cells were fixed on stage by adding 3.7% formaldehyde at 1:1 ratio to culture medium and differential interference contrast images were captured with the × 100 lens and a × 10 0.3 NA lens to mark the position of the cells imaged on the grid (Fig. 10a). Cells were post fixed with 2.5% glutaraldehyde and 4% formaldehyde in 0.1 M phosphate buffer (pH 7.4) for 30 min and then stained, dehydrated and embedded in resin following the National Centre for Microscopy and Imaging Research method ( http://ncmir.ucsd.edu/sbfsem-protocol.pdf). Durcupan-embedded cells were trimmed to a block face of <1 mm^2^, mounted on a stub and imaged in an Auriga Focused Ion Beam SEM (Carl Zeiss). The cell of interest was relocated by referencing the brightfield map and an 80 nm thick layer of platinum was deposited to protect the top face of the block during ion beam milling. Imaging was performed with a backscattered electron detector, at a voltage of 1.65 kV and a 60 μm aperture. Imaging parameters were set at 3072 × 2304 pixels, with a dwell time of 3.23 μsec, a horizontal field width of 18.43 μm and a pixel size of 6 nm. Ion beam milling was performed at a current of 5 nA. The presented SEM stack consists of 867 slices at a slice thickness of 10 nm. The electron microscopy images were aligned using the SIFT linear stack alignment plugin in ImageJ (developed by Stephan Saalfeld^68^). Aligned and rotated electron microscopy stacks were overlaid with light microscopy (LM) stacks using Volocity Image analysis software (Perkin Elmer; Fig. 10e).

### Statistics

Significance levels for comparisons between two groups were determined as stated in corresponding figures legends. *P=0.05%; **P=0.01%; ***P=0.001%; ****P=0.0001%.

### Data availability

The data that support the findings of this study are available from the corresponding author upon request.

## Additional information

**How to cite this article:** Karanasios, E. *et al*. Autophagy initiation by ULK complex assembly on ER tubulovesicular regions marked by ATG9 vesicles. *Nat. Commun.* 7:12420 doi: 10.1038/ncomms12420 (2016).

## Supplementary Material

Supplementary Information Supplementary Figures 1-7
