# Supplementary material for: Autophagy initiation by ULK complex assembly on ER tubulovesicular regions marked by ATG9 vesicles
Source: Nat Commun. 2016 Aug 11;7:12420. doi: 10.1038/ncomms12420 (PMC4987534; doi:10.1038/ncomms12420)
Supplement: Supplementary Information — Supplementary Figures 1-7 [file ncomms12420-s1.pdf]

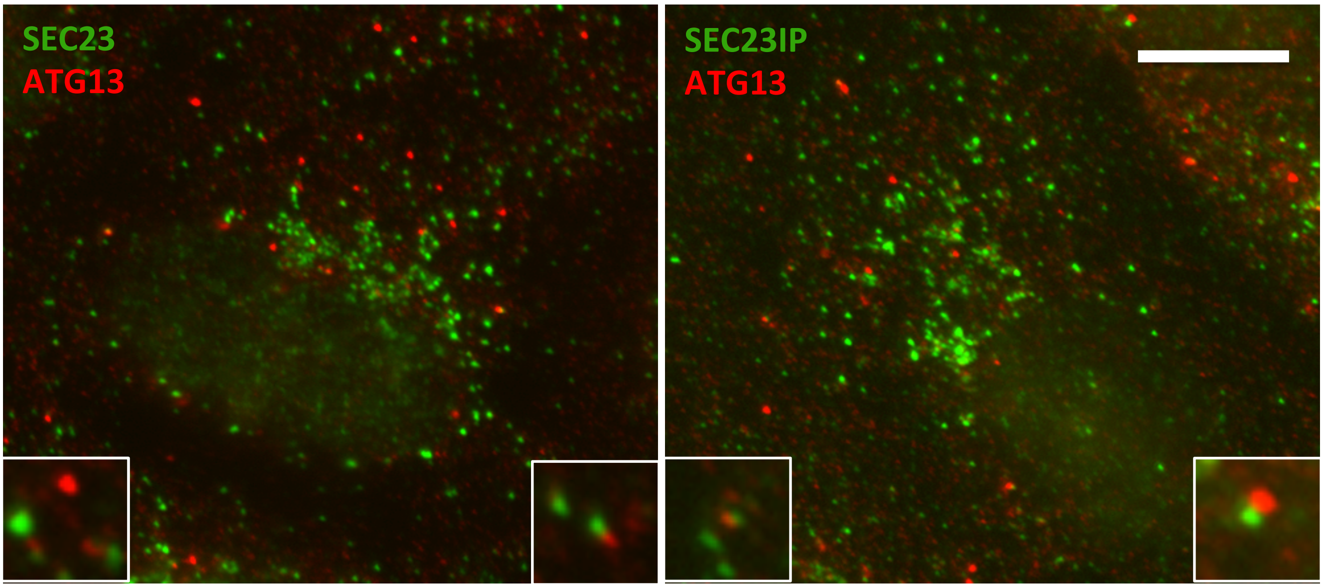

Supplementary figure 1. ATG13 colocalisation with ERES. HEK293 cells were fed or starved for 1 h, immunolabelled for ATG13 and SEC23 or SEC23IP and imaged by widefield microscopy. Bar corresponds to 10 μm.

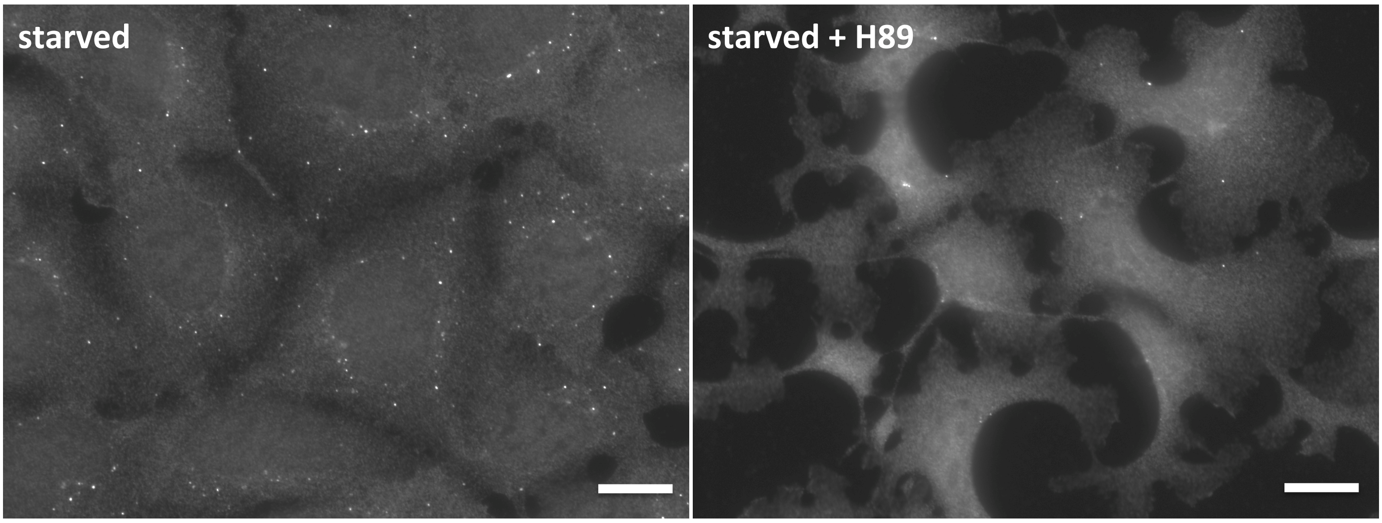

Supplementary figure 2. H89 treatment alters cellular morphology. HEK293 cells were starved for 1 h, treated with 10 μM H89 in the last 30 min, immunolabelled for ATG16 and imaged by widefield microscopy. Bar corresponds to 10 μm.

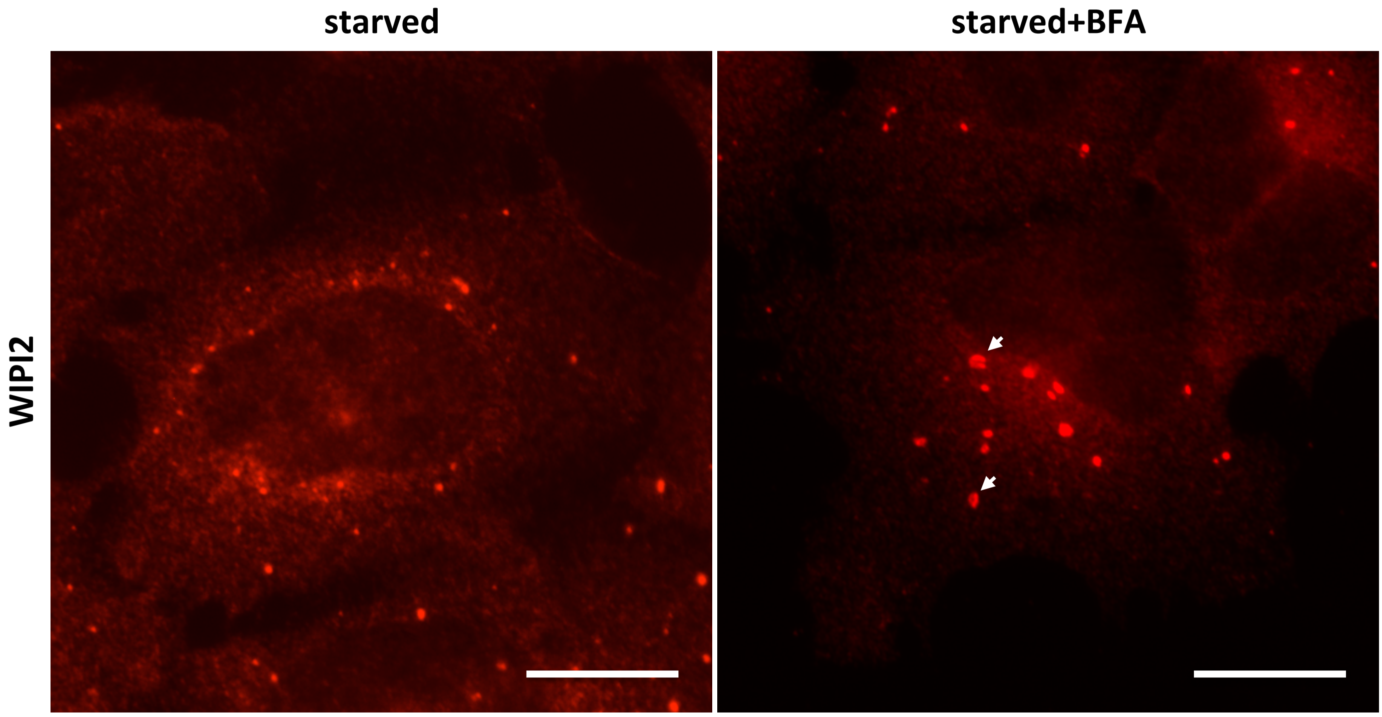

Supplementary figure 3. Brefeldin A treatment misdirects WIPI2 to ring-like structures. HEK293 cells were pre-treated for 3 h with 3 µg ml^{-1} BFA (long BFA), starved for 1 h in the presence of BFA, immunolabelled for WIPI2 and imaged by widefield microscopy. Arrows point at WIPI2 ring-like structures. Bar corresponds to 10 µm.

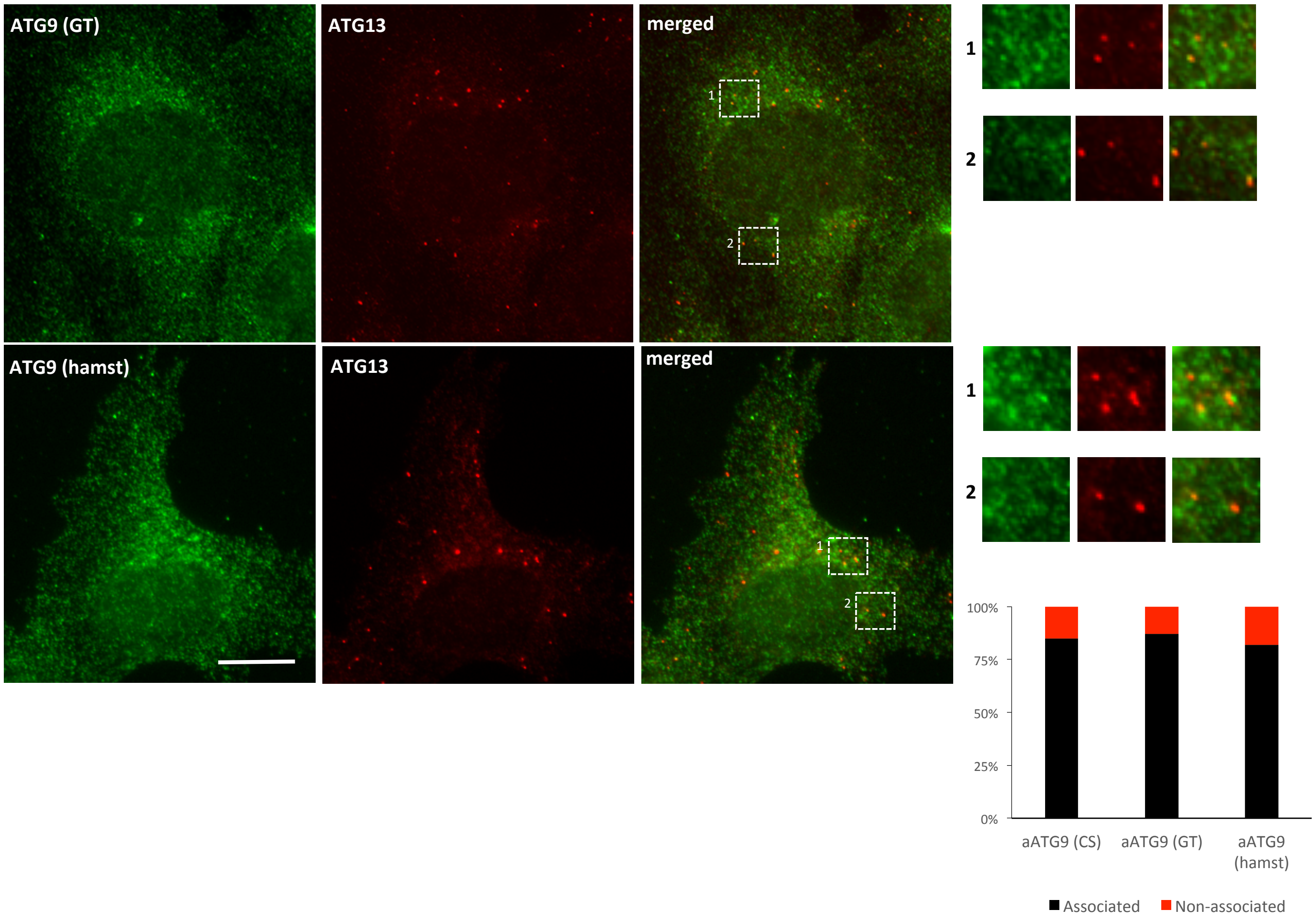

Supplementary figure 4. ATG13 particles colocalise with ATG9. HEK293 cells were starved for 1 h, immunolabelled for ATG13 and ATG9 with 2 different antibodies [from GeneTex (GT) and gift from S. Tooze raised in hamster (hamst)] and imaged by widefield microscopy. Values are ATG13 particles associating with at least one ATG9 vesicle. Values from experiment staining ATG9 with antibody from Cell Signalling (CS, presented in Fig. 4A), are also included. From analysis of 100 ATG13 particles. Bar corresponds to 10 μm.

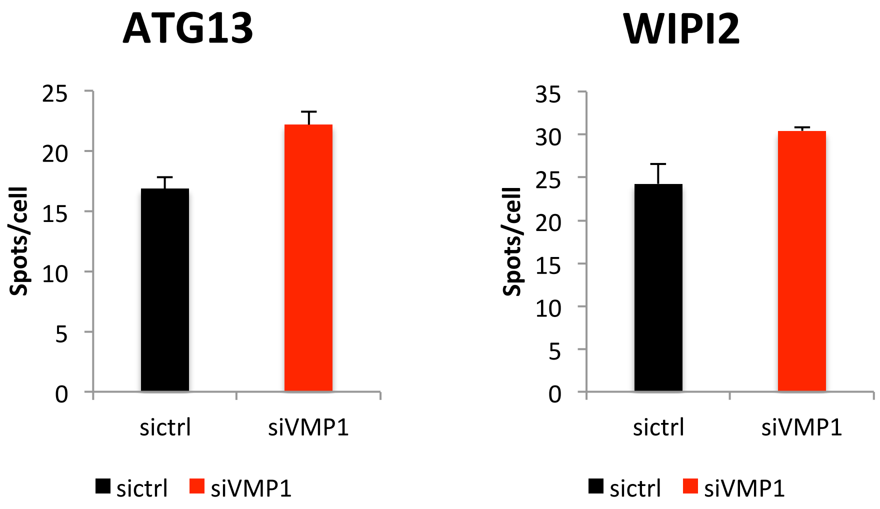

Supplementary figure 5. VMP1 is not required for autophagosome nucleation. HEK293 cells were transfected with non-targeted (siNT) or VMP1 (siVMP1) siRNA, treated for 1 h with 1 µM PP242 (mTOR inhibitor), immunolabelled for ATG13 or WIPI2 and imaged by confocal laser scanning microscopy. Values are means ± SEM puncta of ATG13 or WIPI2 per cell, for 5 different fields with 15–25 cells each.

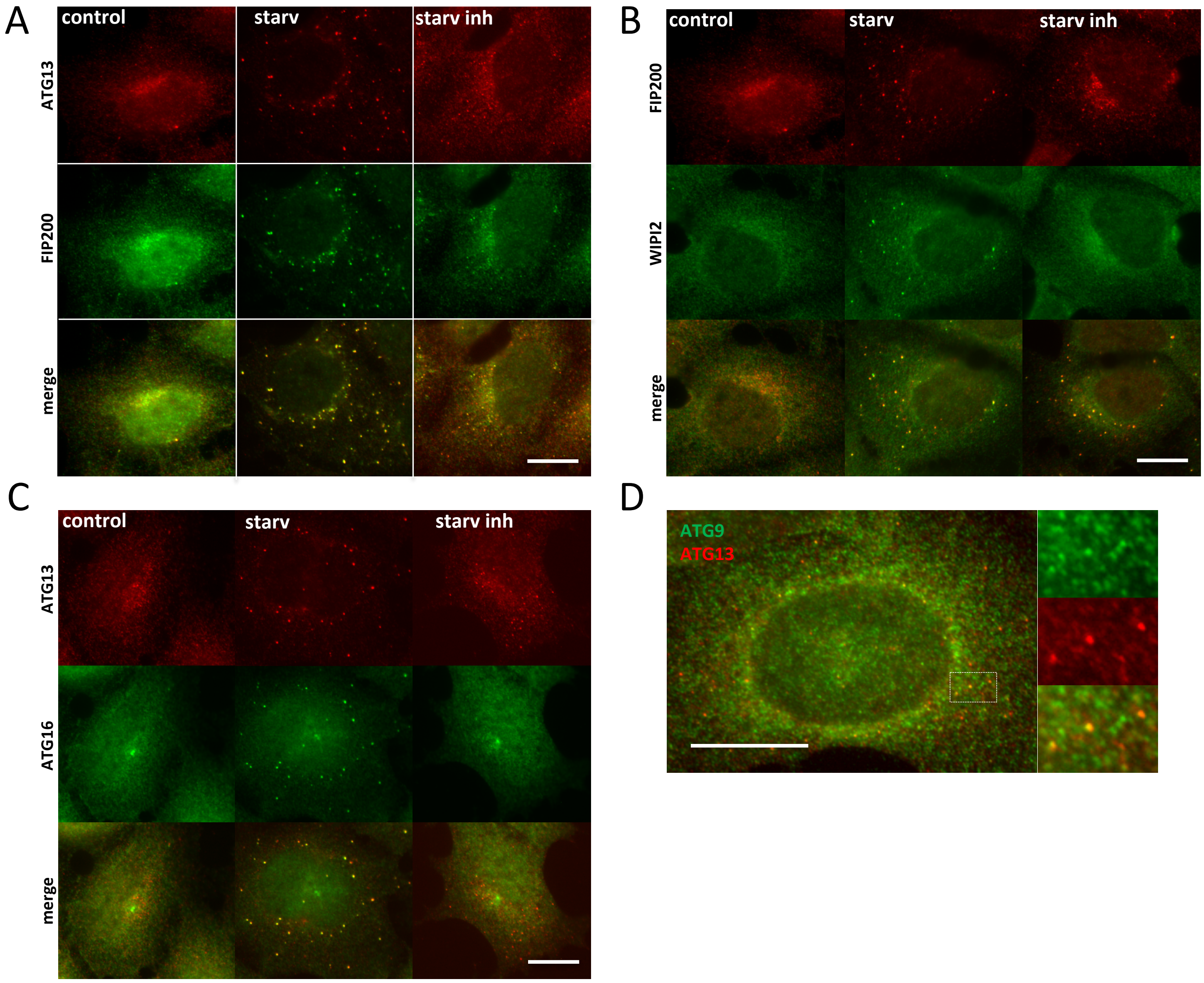

Supplementary figure 6. Characterization of a novel highly selective Vps34 inhibitor. HEK293 cells were fed or starved in the presence or absence of VPS34 inhibitor for 1 h, immunolabelled for ATG13 and FIP200 (A), FIP200 and WIPI2 (B), ATG13 and ATG16 (C) or ATG9 and ATG13 (D) and imaged by widefield microscopy. Bar corresponds to 10 μm.

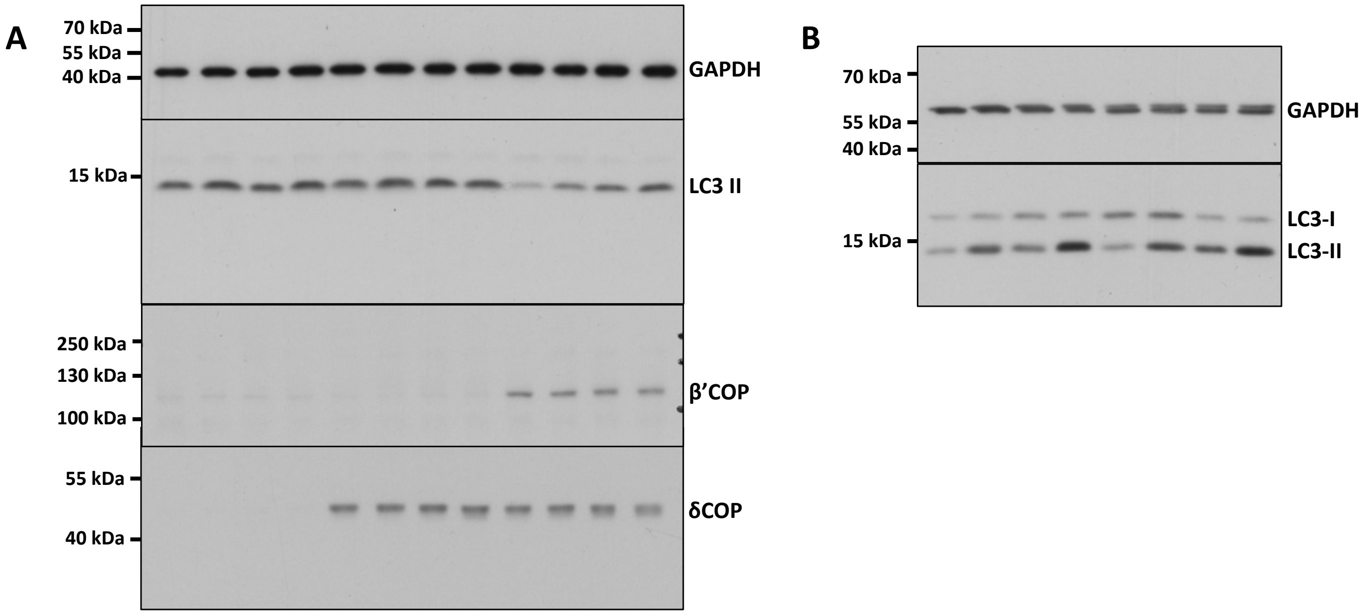

Supplementary figure 7. Western blots in Fig. 2.
